# Genetic deletion of nitric oxide synthase 2 ameliorates Parkinson’s disease pathology and neuroinflammation in a transgenic mouse model of synucleinopathy

**DOI:** 10.1186/s13041-023-00996-1

**Published:** 2023-01-16

**Authors:** Jieun Kim, Jung-Youn Han, Yujeong Lee, Kipom Kim, Young Pyo Choi, Sehyun Chae, Hyang-Sook Hoe

**Affiliations:** 1 Department of Neurodegenerative Diseases Group, Korea Brain Research Institute (KBRI), 61, Cheomdan-Ro, Dong-Gu, Daegu, 41062 South Korea; 2 Laboratory Animal Center, Korea Brain Research Institute (KBRI), 61, Cheomdan-Ro, Dong-Gu, Daegu, 41062 South Korea; 3 Cognitive Science Research Group, Korea Brain Research Institute (KBRI), 61, Cheomdan-Ro, Dong-Gu, Daegu, 41062 South Korea; 4 Research Strategy Office, Korea Brain Research Institute (KBRI), 61, Cheomdan-Ro, Dong-Gu, Daegu, 41062 South Korea; 5 Neurovescular Unit Research Group, Korea Brain Research Institute (KBRI), 61, Cheomdan-Ro, Dong-Gu, Daegu, 41062 South Korea; 6 Department of Brain and Cognitive Sciences, Daegu Gyeongbuk Institute of Science & Technology, Daegu, 42988 South Korea

**Keywords:** α-Synuclein, *nos2*, Neuroinflammation, Parkinson’s disease

## Abstract

Studies of mouse models of Alzheimer's disease (AD) have demonstrated that nitric oxide synthase 2 (NOS2) is involved in AD pathology. However, the effects of NOS2 on the pathology of Parkinson’s disease (PD) are not well studied. To address this gap, we examined the impact of NOS2 on disease-associated phenotypes in a mouse model of PD. Transgenic mice carrying the A53T mutation of α-synuclein (Syn^A53T^) and newly generated double transgenic mice with deletion of NOS2 (Syn^A53T^/NOS2^−/−^) were used. Compared with Syn^A53T^ mice, the loss of *nos2* decreased α-synuclein phosphorylation at serine 129 and reduced α-synuclein-induced microglial and astrocyte activation in Syn^A53T^/NOS^−/−^ mice. Additionally, neuroinflammation-related gene clusters in the deep mesencephalic nucleus (DpMe) were altered in Syn^A53T^/NOS^−/−^ mice compared with Syn^A53T^ mice. Taken together, our results suggest that deletion of *nos2* alleviates α-synuclein pathology and α-synuclein-associated neuroinflammatory responses in the brain.

**Supplementary Information:**

The online version contains supplementary material available at 10.1186/s13041-023-00996-1.

## Main text

Nitric oxide (NO) is a bioactive free radical that is involved in various physiological and pathological processes in several organ systems and the central nervous system (CNS) [1]. In the brain, nitric oxide synthase 2 (NOS2) plays an important role in neurotransmission, neural development, and the immune defense response [2]. Interestingly, several recent studies have reported that NOS2 differentially regulates Alzheimer’s disease (AD) pathology. For instance, deletion of *nos2* in mice results in the expression of mutant amyloid precursor protein (APP) and hyperphosphorylation of tau in the brain [3]. Compared with APPSwDI mice, APPSwDI/NOS2^−/−^ mice exhibit spatial memory impairment and tau pathology [4]. However, the effects of NOS2 on α-synuclein-induced Parkinson’s disease (PD) pathology remain unclear.

To address this gap, we generated Syn^A53T^/NOS2^−/−^ mice by hybridizing human Syn^A53T^-expressing transgenic mice and *nos2* knockout (NOS2^−/−^) mice. Generation of the Syn^A53T^/NOS2^−/−^ mice was confirmed by RT–PCR, which failed to detect *nos2* mRNA (Additional file 1: Fig. S1).

**Fig. 1:**
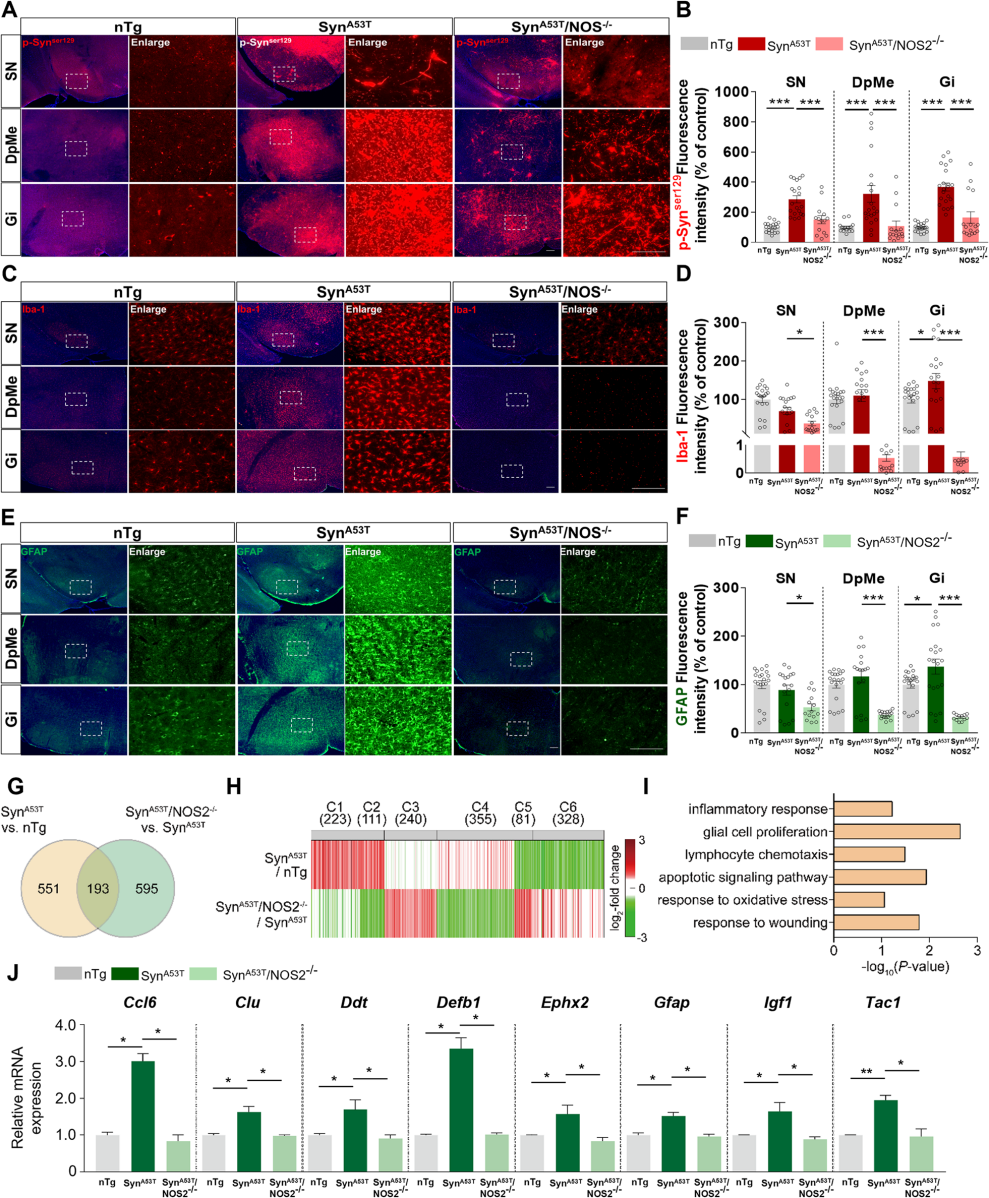
Deletion of *nos2* alleviates synuclein pathology and neuroinflammatory responses in Syn^A53T^/NOS2^−/−^ mice. **A** Immunofluorescence staining of the substantia nigra (SN), deep mesencephalic nucleus (DpMe), and granular insular cortex (Gi) of 10- to 11-month-old nTg, Syn^A53T^, and Syn^A53T^/NOS2^−/−^ mice with an anti-p-Syn^ser129^ antibody. **B** Quantification of the data in A (SN, DpMe, Gi region; nTg: n = 17–20 brain slices/5 mice; Syn^A53T^: n = 20–22 brain slices/5 mice; Syn^A53T^/NOS2^−/−^: n = 14–16 brain slices/4 mice). **C** Immunofluorescence staining of the SN, DpMe, and Gi of 10- to 11-month-old nTg, Syn^A53T^, and Syn^A53T^/NOS2^−/−^ mice with an anti-Iba-1 antibody. **D** Quantification of the data in C (SN, DpMe, Gi region; nTg: n = 20 brain slices/5 mice; Syn^A53T^: n = 17–20 brain slices/5 mice; Syn^A53T^/NOS2^−/−^: n = 11–15 brain slices/4 mice). **E** Immunofluorescence staining of the SN, DpMe, and Gi of 10- to 11-month-old nTg, Syn^A53T^, and Syn^A53T^/NOS2^−/−^ mice with an anti-GFAP antibody. **F** Quantification of the data in E (SN, DpMe, Gi region; nTg: n = 20 brain slices/5 mice; Syn^A53T^: n = 17–20 brain slices/5 mice; Syn^A53T^/NOS2^−/−^: n = 12–16 brain slices/4 mice). **G** Differentially expressed genes (DEGs) were identified by comparing Syn^A53T^/NOS2^−/−^ mice with Syn^A53T^ mice and Syn^A53T^ mice with nTg mice. The numbers of DEGs in each comparison and the number of overlapping DEGs are indicated. **H** Six clusters (C1–6) of DEGs were identified from the two comparisons. The color bar represents the gradient of log_2_ fold changes. The number of DEGs in each cluster is denoted in parentheses. **I** Cellular processes represented by the DEGs in C2. The x-axis is the −log_10_(*P*), where *P* is the enrichment *P* value from ConsensusPathDB software. **J** DEGs involved in the inflammatory response (n = 3 mice/group). *p < 0.05, **p < 0.01, ***p < 0.001, scale bar = 100 μm

We then examined whether genetic deletion of *nos2* affects α-synuclein-induced PD pathology. The brains of 10- to 11-month-old non-transgenic (nTg), Syn^A53T^, and Syn^A53T^/NOS2^−/−^ mice were subjected to immunofluorescence staining with an anti-p-Syn^ser129^ antibody. Compared with nTg mice, p-Syn^ser129^ levels in the substantia nigra (SN), deep mesencephalic reticular nucleus (DpMe), and granular insular cortex (Gi) were significantly higher in Syn^A53T^ mice (Fig. 1A, B). Importantly, p-Syn^ser129^ levels in the SN, DpMe, and Gi were significantly lower in Syn^A53T^/NOS2^−/−^ mice than in Syn^A53T^ mice (Fig. 1A, B). Moreover, p-Syn^ser129^ levels in the cortex, caudate and putamen (CPu) and hippocampus (Hippo) were significantly reduced in Syn^A53T^/NOS2^−/−^ mice compared with Syn^A53T^ mice (Additional file 1: Fig. S2A, B). These data suggest that genetic deletion of *nos2* alleviates synucleinopathy in the brain.

Since genetic deletion of *nos2* diminished α-synuclein aggregation in the brain, we further investigated the impact of *nos2* deletion on α-synuclein-induced glial activation. The brains of 10- to 11-month-old nTg, Syn^A53T^, and Syn^A53T^/NOS2^−/−^ mice were subjected to immunofluorescence staining with anti-Iba-1 and anti-GFAP antibodies. Compared with nTg mice, microglial/astrocyte fluorescence intensity, the number of Iba-1-positive cells, and the Iba-1/GFAP % area were increased in the DpMe and Gi but not in the SN in Syn^A53T^ mice (Fig. 1C, D and Additional file 1: Fig. S3). Importantly, Iba-1 fluorescence intensity, the number of Iba-1-positive cells, and the Iba-1-positive % area in the SN, DpMe, and Gi were significantly lower in Syn^A53T^/NOS2^−/−^ mice than in Syn^A53T^ mice (Fig. 1C, D and Additional file 1: Fig. S3). Moreover, GFAP fluorescence intensity in the SN, DpMe, and Gi was significantly reduced in Syn^A53T^/NOS2^−/−^ mice compared with Syn^A53T^ mice (Fig. 1E, F). The α-synuclein-induced number of GFAP-positive cells and GFAP-positive % area in the DpMe and Gi were significantly diminished in Syn^A53T^/NOS2^−/−^ mice compared with Syn^A53T^ mice (Additional file 1: Fig. S3). In addition, Iba-1/GFAP fluorescence intensity, the number of Iba-1/GFAP-positive cells and the Iba-1/GFAP % area in the cortex, CPu, and hippocampus were significantly reduced in Syn^A53T^/NOS2^−/−^ mice compared with nTg and Syn^A53T^ mice (Figs. S4-S5). Taken together, these data suggest that deletion of *nos2* diminishes α-synuclein-stimulated microglial and astrocyte activation and that NOS2 is required for α-synuclein-mediated neuroinflammation in the brain.

To investigate the effects of *nos2* deletion on gene expression in the mouse model of PD, we isolated the DpMe region (which exhibited the greatest regulatory effects of *nos2*) from 10- to 11-month-old nTg, Syn^A53T^, and Syn^A53T^/NOS2^−/−^ mice and conducted RNA sequencing. A total of 1,339 differentially expressed genes (DEGs) were identified in Syn^A53T^ versus nTg mice and Syn^A53T^/NOS2^−/−^ versus Syn^A53T^ mice (744 and 788 DEGs, respectively) (Fig. 1G and Additional file 2: Table S1). Among the 1339 DEGs, 193 overlapped between the two comparisons (Fig. 1G). These results indicate that *nos2* deletion significantly alters gene expression in this mouse model of PD (Additional file 3).

To systematically investigate the cellular processes affected by *nos2* deletion, we classified the 1,339 DEGs into 6 clusters (C1-6) based on their differential expression in the two comparisons (Fig. 1H). C2 was upregulated in Syn^A53T^ mice compared with nTg mice but downregulated in Syn^A53T^/NOS2^−/−^ mice compared with Syn^A53T^ mice. Thus, we focused on this cluster because it likely includes genes associated with the effects of NOS2 on PD pathology. The cellular processes represented by the DEGs in C2 were identified by gene set enrichment analysis using Consensus Path DB [5]. Interestingly, the DEGs in C2 were mainly involved in neuroinflammatory responses, glial cell proliferation, oxidative stress, and apoptosis (Fig. 1I). Notably, genes involved in neuroinflammatory response-related processes were strongly downregulated in Syn^A53T^/NOS2^−/−^ mice compared with Syn^A53T^ mice (Fig. 1J).

In summary, α-synuclein phosphorylation, α-synuclein-induced neuroinflammation, and the expression of related genes were significantly suppressed in the brains of Syn^A53T^/NOS2^−/−^ mice. Overall, our results suggest that NOS2 is a crucial regulator of the synucleinopathy and neuroinflammatory response associated with PD pathology.

A recent study demonstrated that NOS2 overexpression induces NO production and α-synuclein aggregation in PC12 neurons [6]. In SH-SY5Y cells, NOS2 expression induces the formation of cytotoxic nitrated α-synuclein [7]. However, the effects of *nos2* deletion on α-synuclein pathology have not been investigated. The significant reduction in p-Syn^ser129^ levels in Syn^A53T^/NOS2^−/−^ mice compared with Syn^A53T^ mice suggests that decreasing NOS2 expression may help alleviate α-synucleinopathy in the brain.

Interestingly, several recent studies have shown that NOS2 regulates neuroinflammatory responses in the brain. For instance, the lipopolysaccharide (LPS)-induced increase in TNF-α levels is significantly reduced in *nos2* knockout mice [8], and deletion of *nos2* decreases the number of Iba-1/GFAP-positive cells in the brain compared with wild-type mice [9]. In addition, GFAP expression is diminished by one-third in NOS2^−/−^ mice compared with nTG mice [10]. In the present study, microglial and astrocyte activation in the brain, which are associated with severe synuclein pathology, were dramatically reduced in Syn^A53T^/NOS2^−/−^ mice compared with Syn^A53T^ mice. It is possible that brain region-specific synuclein aggregation and pathology contribute to Iba-1/GFAP expression when *nos2* is knocked out. Another possibility is that unknown synuclein pathology/NOS2-associated molecular targets contribute to glial hypoactivity/degradation when *nos2* is deleted in vivo. Future studies will focus on identifying the molecules that contribute to glial inactivation and the amelioration of synuclein pathology when *nos2* is deleted. Overall, the available data suggest that NOS2 has critical functions in the modulation of glial homeostasis in this mouse model of PD.

In conclusion, we generated Syn^A53T^/NOS2^−/−^ mice for the first time by crossing human α-synuclein A53T mutant mice and *nos2* knockout mice and found that α-synuclein pathology, neuroinflammatory responses, and neuroinflammation-associated gene expression were reduced in the double transgenic mice compared with Syn^A53T^ mice. Our data indicate that NOS2 may be a therapeutic target for modulating PD pathology in the brain.

## Supplementary Information

**Additional file 1: Figure S1.** The *nos2* mRNA expression was not detected in Syn^A53T^/NOS2^−/−^ mice. **Figure S2.** The p-Syn^Ser129^ levels in the cortex, caudate and putamen, and hippocampus were significantly diminished in Syn^A53T^/NOS2^−/−^ mice compared with Syn^A53T^ mice. **Figure S3.** The number of Iba-1/GFAP-positive cells and % area fractions in the substantia nigra, deep mesencephalic nucleus, and granular insular cortex were significantly reduced in Syn^A53T^/NOS2^−/−^ mice compared with Syn^A53T^ mice. **Figure S4.** The Iba-1-fluorescence intensity, number of Iba-1-positive cells, and % area fractions in the cortex, caudate and putamen, and hippocampus were significantly suppressed in Syn^A53T^/NOS2^−/−^ mice compared with Syn^A53T^ mice. **Figure S5.** The GFAP-fluorescence intensity, number of GFAP-positive cells, and % area fractions in the cortex, caudate and putamen, and hippocampus were significantly downregulated in Syn^A53T^/NOS2^−/−^ mice compared with Syn^A53T^ mice. **Materials and methods.**

**Additional file 2: Table S1.** The lists of the 1,339 DEGs included in the individual clusters.

**Additional file 3: Table S2.** One-way ANOVA (Tukey’s test) and significance of the results of the in vivo experiments in this study.

## Data Availability

All data generated and/or analyzed during this study are included in this published article and its supplementary information. The materials and methods are presented in Additional file 1.

## Abbreviations

AD: Alzheimer’s disease
CPu: Caudate and putamen
DEGs: Differentially expressed genes
DpMe: Deep mesencephalic reticular nucleus
Gi: Granular insular cortex
Hippo: Hippocampus
LPS: Lipopolysaccharide
NOS2: Nitric oxide synthase 2
PD: Parkinson’s disease
SN: Substantia nigra
