# Supplementary material for: Genetic deletion of nitric oxide synthase 2 ameliorates Parkinson’s disease pathology and neuroinflammation in a transgenic mouse model of synucleinopathy
Source: Mol Brain. 2023 Jan 16;16:7. doi: 10.1186/s13041-023-00996-1 (PMC9841612; doi:10.1186/s13041-023-00996-1)
Supplement: Supplementary file 1 — Additional file 1: Figure S1. The nos2 mRNA expression was not detected in SynA53T/NOS2−/− mice. Figure S2. The p-SynSer129 levels in the cortex, caudate and putamen, and hippocampus were significantly diminished in SynA53T/NOS2−/− mice compared with SynA53T mice. Figure S3. The number of Iba-1/GFAP-positive cells and % area fractions in the substantia nigra, deep mesencephalic nucleus, and granular insular cortex were significantly reduced in SynA53T/NOS2−/− mice compared with SynA53T mice. Figure S4. The Iba-1-fluorescence intensity, number of Iba-1-positive cells, and % area fractions in the cortex, caudate and putamen, and hippocampus were significantly suppressed in SynA53T/NOS2−/− mice compared with SynA53T mice. Figure S5. The GFAP-fluorescence intensity, number of GFAP-positive cells, and % area fractions in the cortex, caudate and putamen, and hippocampus were significantly downregulated in SynA53T/NOS2−/− mice compared with SynA53T mice. Materials and methods. [file 13041_2023_996_MOESM1_ESM.docx]

**Genetic deletion of nitric oxide synthase 2 ameliorates Parkinson’s disease pathology and neuroinflammation in a transgenic maouse model of synucleinopathy**

Jieun Kim^1,+^, Jung-Youn Han^2,+^, Yujeong Lee^3^, Kipom Kim^4^, Young Pyo Choi^2,*^, Sehyun Chae^5,*^, Hyang-Sook Hoe^1,6,*^

^1^Department of Neurodegenerative Disease, ^2^Laboratory Animal Center, ^3^Cognitive Science Research Group, ^4^Research Strategy Office, and ^5^Neurovescular Unit Research Group, Korea Brain Research Institute (KBRI), 61, Cheomdan-ro, Dong-gu, Daegu, Korea, 41062; ^6^Department of Brain and Cognitive Sciences, Daegu Gyeongbuk Institute of Science & Technology, Daegu, Korea 42988. ^+^ These authors contributed equally to this work.

^*^Corresponding author:

Hyang-Sook Hoe, Ph.D., Neurodegenerative Diseases Group, Korea Brain Research Institute (KBRI), 61, Cheomdan-ro, Dong-gu, Daegu, Korea, 41062; E-mail: [*sookhoe72@kbri.re.kr*](mailto:sookhoe72@kbri.re.kr)

Sehyun Chae, Ph.D., Neurovascular Unit Research Group, Korea Brain Research Institute (KBRI), 61, Cheomdan-ro, Dong-gu, Daegu, Korea, 41062; E-mail: *shchae@kbri.re.kr*

Young Pyo Choi, D.V.M., Ph.D., Laboratory Animal Center, Korea Brain Research Institute (KBRI), 61, Cheomdan-ro, Dong-gu, Daegu, Korea, 41062; E-mail: *cyp0201@kbri.re.kr*

**
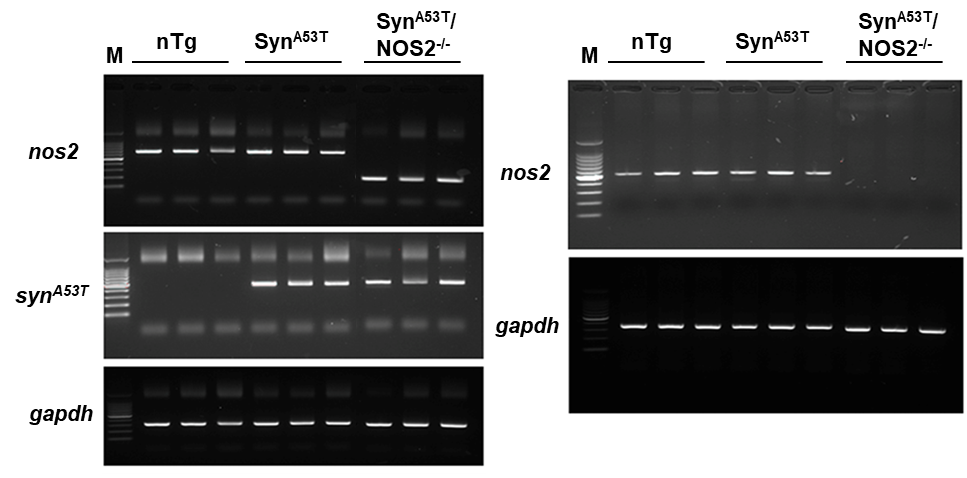
**

**Additional file 1: Figure S1.** The *nos2* mRNA expression was not detected in Syn^A53T^/NOS2^-/-^ mice. RT–PCR analysis of brain tissue from 10- to 11-month-old nTg, Syn^A53T^, and Syn^A53T^/NOS2^-/-^ mice was performed using primers for *nos2* (n = 3 mice/group).

**
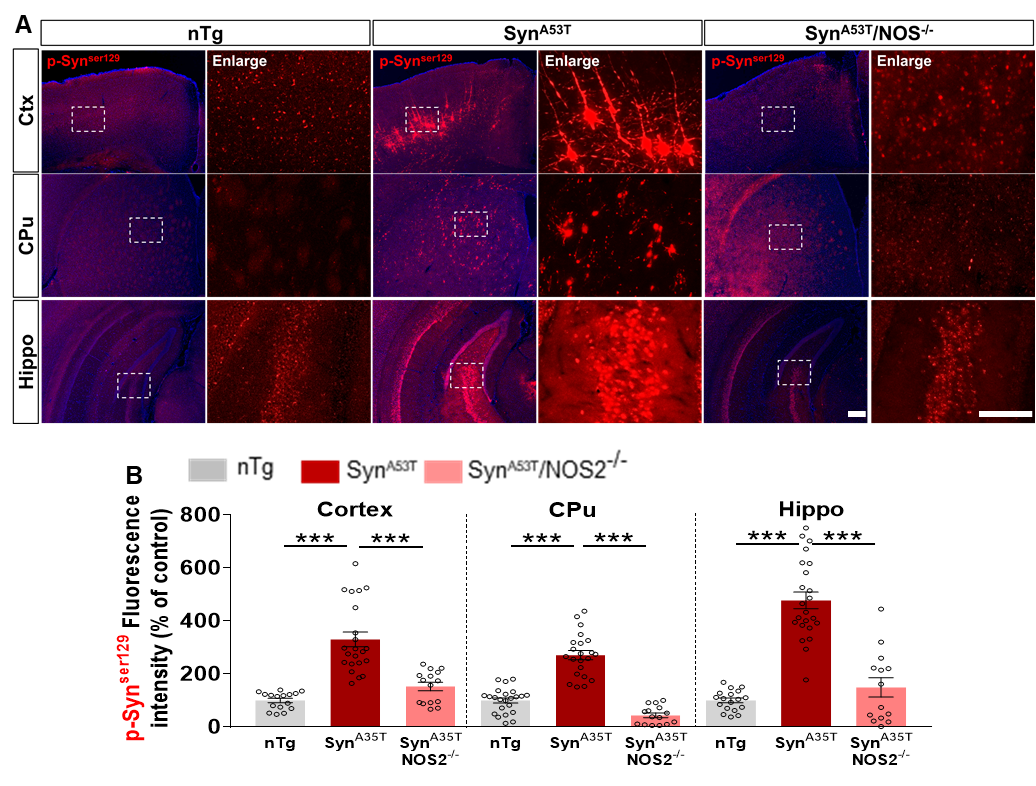
**

**Additional file 1: Figure S2.** The p-Syn^Ser129^ levels in the cortex, caudate and putamen, and hippocampus were significantly diminished in Syn^A53T^/NOS2^-/-^ mice compared with Syn^A53T^ mice. (A) Immunofluorescence staining of the brains of 10- to 11-month-old nTg, Syn^A53T^, and Syn^A53T^/NOS2^-/-^ mice was conducted with an anti-p-Syn^Ser129^ antibody. (B) Quantification of the data in A (Cortex, CPu, Hipp region; nTg: n = 16-20 brain slices/5 mice; Syn^A53T^: n = 22-23 brain slices/5 mice; Syn^A53T^/NOS2^-/-^: n = 14-15 brain slices/4 mice). *** *P* < 0.001, scale bar = 100 μm, CPu: caudate and putamen; Hippo: hippocampus.

**
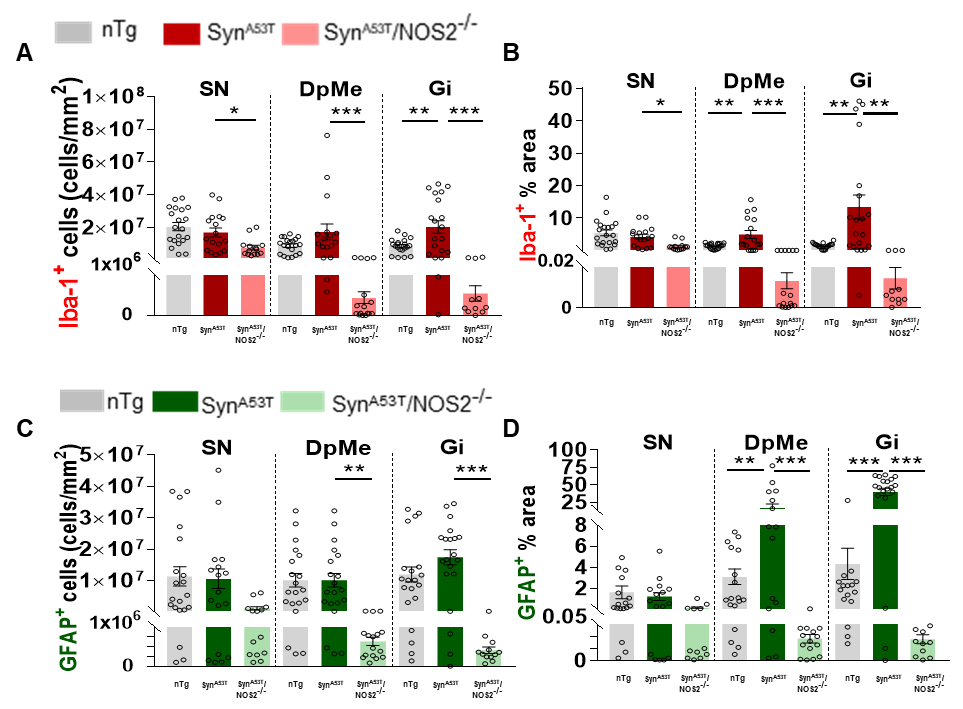
**

**Additional file 1: Figure S3.** The number of Iba-1/GFAP-positive cells and % area fractions in the substantia nigra, deep mesencephalic nucleus, and granular insular cortex were significantly reduced in Syn^A53T^/NOS2^-/-^ mice compared with Syn^A53T^ mice. (A-D) Quantification of the immunofluorescence staining of the brains of 10- to 11-month-old nTg, Syn^A53T^, and Syn^A53T^/NOS2^-/-^ mice with anti-Iba-1 and anti-GFAP antibodies (SN, DpMe, Gi region; nTg: n = 20 brain slices/5 mice; Syn^A53T^: n = 17-20 brain slices/5 mice; Syn^A53T^/NOS2^-/-^: n = 11-16 brain slices/4 mice). * *P* < 0.05, ** *P* < 0.01, *** *P* < 0.001, SN: substantia nigra; DpMe: deep mesencephalic nucleus; Gi: granular insular cortex.

**
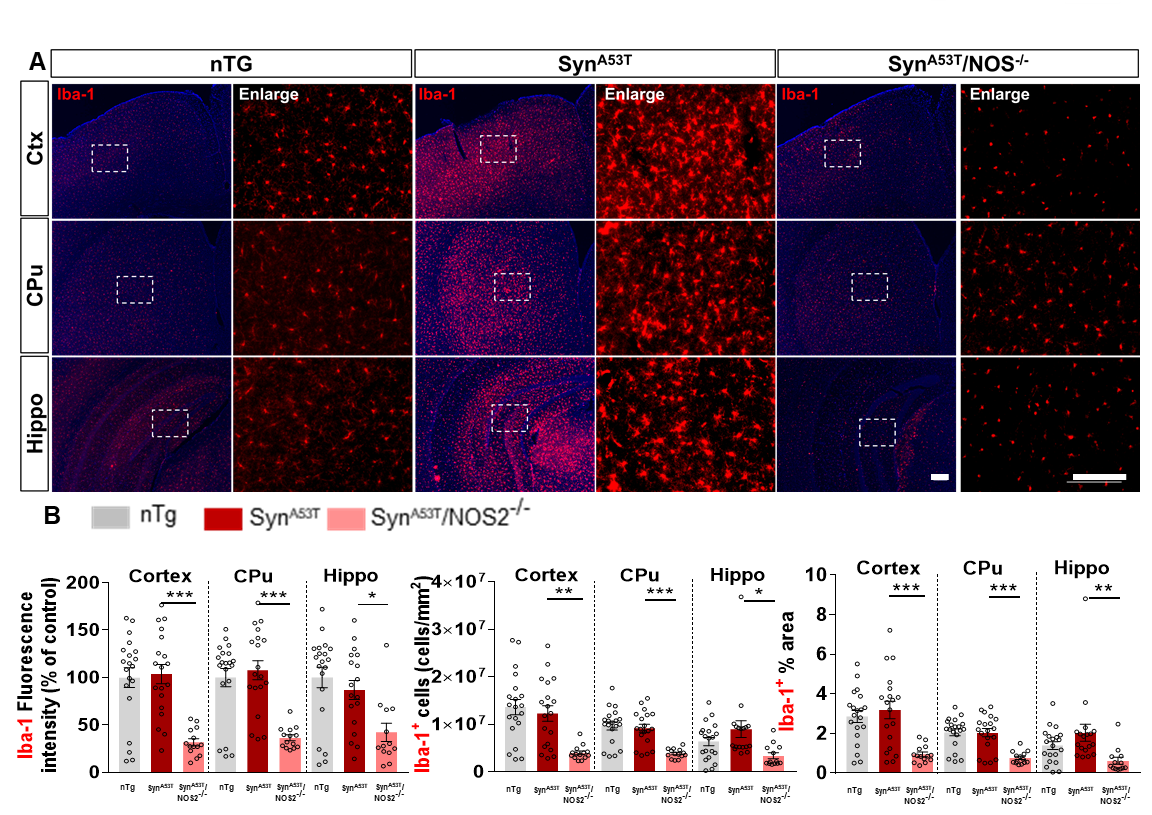
**

**Additional file 1: Figure S4.** The Iba-1-fluorescence intensity, number of Iba-1-positive cells, and % area fractions in the cortex, caudate and putamen, and hippocampus were significantly suppressed in Syn^A53T^/NOS2^-/-^ mice compared with Syn^A53T^ mice. (A-B) Quantification of the immunofluorescence staining of the brains of 10- to 11-month-old nTg, Syn^A53T^, and Syn^A53T^/NOS2^-/-^ mice with an anti-Iba-1 antibody (Cortex, CPu, Hippo region; nTg: n = 20 brain slices/5 mice; Syn^A53T^: n = 18-19 brain slices/5 mice; Syn^A53T^/NOS2^-/-^: n = 13-14 brain slices/4 mice). Scale bar = 100 μm, * *P* < 0.05, ** *P* < 0.01, *** *P* < 0.001, CPu: caudate and putamen; Hippo: hippocampus.

**
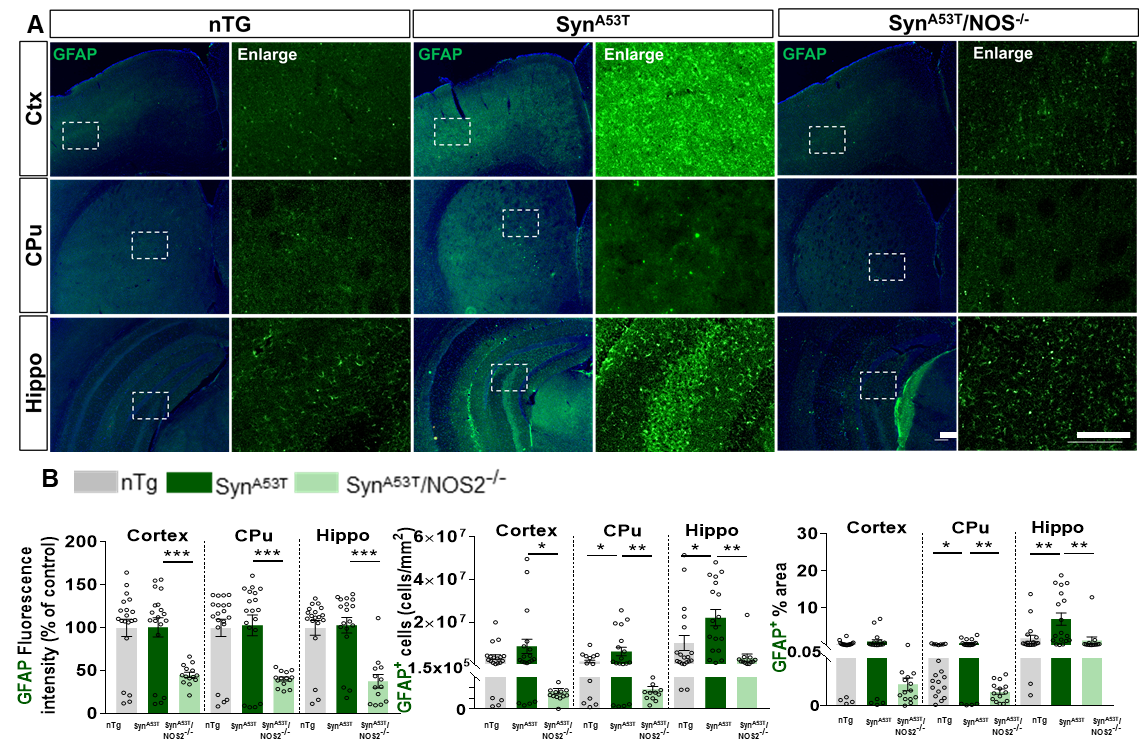
**

**Additional file 1: Figure S5.** The GFAP-fluoresscence intensity, number of GFAP-positive cells, and % area fractions in the cortex, caudate and putamen, and hippocampus were significantly downregulated in Syn^A53T^/NOS2^-/-^ mice compared with Syn^A53T^ mice. (A-B) Quantification of the immunofluorescence staining of the brains of 10- to 11-month-old nTg, Syn^A53T^, and Syn^A53T^/NOS2^-/-^ mice with an anti-GFAP antibody (Cortex, CPu, Hippo region; nTg: n = 20 brain slices/5 mice; Syn^A53T^: n = 18-19 brain slices/5 mice; Syn^A53T^/NOS2^-/-^: n = 13-14 brain slices/4 mice). Scale bar = 100 μm, * *P* < 0.05, ** *P* < 0.01, *** *P* < 0.001, CPu: caudate and putamen; Hippo: hippocampus.

**2. Materials and methods**

*2.1. Generation of double transgenic mice*

The effects of *nos2* on synuclein pathology and neuroinflammatory responses were assessed in nTg, Syn^A53T^, and Syn^A53T^/NOS2^-/-^ mice. Syn^A53T^ mice expressing human Syn^A53T^ under the control of the murine prion promoter were described previously (1) and were purchased from Jackson Laboratory (Stock No. 006823). The Syn^A53T^ mice were maintained as hemizygotes. NOS2^-/-^ mice lacking NOS2 were reported previously (2) and were also obtained from Jackson Laboratory (Stock No. 002609). Syn^A53T^/NOS2^-/-^ transgenic mice were produced by crossbreeding Syn^A53T^ mice and NOS2^-/-^ mice. All experiments involving mice were performed in accordance with animal protocols approved by the Institutional Animal Care and Use Committee (IACUC) of the Korea Brain Research Institute (KBRI) (IACUC-18-0007 and IACUC-19-0010). The mice were maintained under specific pathogen-free (SPF) conditions with water and food *ad libitum* and a 12-h light/dark cycle. All mice were genotyped by standard PCR using genomic DNA extracted from tail clips.

*2.2. RT–PCR*

Total RNA was extracted from the brainstem and midbrain using an RNeasy Mini Kit (Qiagen, Cat no. 74104) and reverse transcribed into cDNA for use in RT–PCR (Superscript IV First-Strand Synthesis System, Invitrogen). After electrophoretic separation on 1.5% agarose gels with SYBR Safe DNA gel stain (Invitrogen, Cat no. S33102, 1:10,000), images of the RT–PCR products were analyzed using a ChemiDoc^TM^ XRS+ imaging system (Bio-Rad). The expression of *nos2* mRNA was identified using primers as described previously (3).

*2.3. Immunohistochemistry*

At 10 to 11 months of age, the mice were transcardially perfused with PBS followed by 4% paraformaldehyde (PFA) in PBS. The brains were recovered, fixed overnight in 4% PFA, and cryoprotected by consecutive overnight incubations in 15% sucrose in PBS and 30% sucrose in PBS. The brains were frozen, embedded in optimal cutting temperature (OCT) compound in liquid nitrogen, and stored at -80°C until use. OCT brain blocks from nTg, Syn^A53T^ mice and Syn^A53T^/NOS2^-/-^ mice were coronally sectioned at a thickness of 30 μm and immunostained as described previously (4) with modifications. Briefly, free-floating sections were blocked with 10% normal goat serum in PBS plus 0.3% Triton X-100. Subsequently, the sections were incubated with primary antibodies diluted in PBS at 4°C overnight. The primary antibodies included rabbit anti-p-Syn^Ser129^ antibody (1:250, Abcam, ab51253), rabbit anti-Iba-1 antibody (1:500, 019-19741, Wako), and chicken anti-GFAP antibody (1:500, AB5541, Millipore). The sections were then incubated with Alexa Fluor 488- or 555-conjugated secondary antibodies as dictated by the primary antibody host (1:500, Life Technologies) in PBS plus 0.3% Triton X-100 for 2 h at room temperature and mounted with ProLong Gold Antifade with DAPI (Cat. No. D1306; Invitrogen). Images of the stained sections were obtained by scanning with a DMi8 fluorescence microscope (Leica Microsystems, Wetzlar, Germany).

*2.4. mRNA sequencing and data analysis*

Total RNA was obtained from the deep mesencephalic reticular nucleus (DpMe) region of nTg, Syn^A53T^, and Syn^A53T^/NOS2^-/-^ mice. A 2100 Bioanalyzer (Agilent Technologies, Santa Clara, CA, USA) was used to assess RNA integrity. All samples had RNA integrity number (RIN) values > 7. Poly(A) mRNA isolation from the total RNA and subsequent fragmentation were performed using the TruSeq Stranded mRNA LT Sample Prep Kit (Illumina, San Diego, CA, USA) according to the manufacturer’s instructions. An Illumina NovaSeq 6000 (Macrogen Inc., Seoul, South Korea) was used for mRNA sequencing of adaptor-ligated libraries; three biological replicates were sequenced for each condition. The software cutadapt (version 2.7) was used to remove adapter sequences (TruSeq universal and indexed adapters) from the reads, which were then aligned to the *Mus musculus* reference genome (GRCm38) using TopHat2 software (version 2.1.1) with default parameters (5). The numbers of reads mapped to gene features (GTF file of GRCm38.91) were determined using HTSeq (6) and normalized using TMM (trimmed mean of M-values) in the edgeR package (7).

*2.5. Differentially expressed gene identification*

The read counts for the gene features were converted to log_2_-values after adding one (pseudo count) to the read counts. Differentially expressed genes (DEGs) were identified using a statistical hypothesis test as previously reported (8). Briefly, for each gene, a t-statistic value was calculated using Student’s t test in the two comparisons (Syn^A53T^ versus nTg or Syn^A53T^/NOS2^-/-^ versus Syn^A53T^). The samples were then randomly permutated in all possible combinations to estimate the empirical distribution of the t-statistic value for the null hypothesis (i.e., not differentially expressed). The empirical null distribution was used in the two-tailed Student’s t test to calculate adjusted *P* values. Genes with adjusted *P* values < 0.1 and log_2_-fold changes larger than the specified cutoff (0.441; 1.357-fold change) and within the 0.5^th^ and 99.5^th^ percentiles of the null distribution of log_2_-fold changes were identified as DEGs. According to the selection criteria, DEGs were identified from each of the two comparisons (Syn^A53T^ versus nTg or Syn^A53T^/NOS2^-/-^ versus Syn^A53T^). The DEGs were then sorted according to their up-regulation, no change, and down-regulation patterns in the order of the two comparisons. Each DEG was assigned to one of six clusters (C1-6) based on their differential expression in the two comparisons: C1 and C6 included the genes with the up- and down-regulation in Syn^A53T^ versus nTg and no change in Syn^A53T^/NOS2^-/-^ versus Syn^A53T^. C3 and C4 included the genes with no change in Syn^A53T^ versus nTg and up- and down-regulation in Syn^A53T^/NOS2^-/-^ versus Syn^A53T^. C2 and C5 included the genes that changed in opposite directions in the two comparisons. Gene set enrichment analysis of the DEGs was performed using ConsensusPathDB software (version 35) (9). GO biological processes and pathways with *P* < 0.1 were considered enriched in DEGs.

*2.6. Statistical analysis*

All data were analyzed using one-way ANOVA with GraphPad Prism 7 software. Tukey’s multiple comparison test was used for post hoc analysis with significance at *P* < 0.05. Data are presented as the mean ± S.E.M. (**p* < 0.05, ***p* < 0.01, ****p* < 0.001). Detailed information is listed in Additional file 3: Tables S2.

**References**

1. Lee MK, Stirling W, Xu Y, Xu X, Qui D, Mandir AS, et al. Human alpha-synuclein-harboring familial Parkinson's disease-linked Ala-53 --> Thr mutation causes neurodegenerative disease with alpha-synuclein aggregation in transgenic mice. Proc Natl Acad Sci U S A. 2002;99(13):8968-73.

2. Laubach VE, Shesely EG, Smithies O, Sherman PA. Mice lacking inducible nitric oxide synthase are not resistant to lipopolysaccharide-induced death. Proc Natl Acad Sci U S A. 1995;92(23):10688-92.

3. Colton CA, Vitek MP, Wink DA, Xu Q, Cantillana V, Previti ML, et al. NO synthase 2 (NOS2) deletion promotes multiple pathologies in a mouse model of Alzheimer's disease. Proc Natl Acad Sci U S A. 2006;103(34):12867-72.

4. Ryu KY, Lee HJ, Woo H, Kang RJ, Han KM, Park H, et al. Dasatinib regulates LPS-induced microglial and astrocytic neuroinflammatory responses by inhibiting AKT/STAT3 signaling. J Neuroinflammation. 2019;16(1):190.

5. Trapnell C, Pachter L, Salzberg SL. TopHat: discovering splice junctions with RNA-Seq. Bioinformatics. 2009;25(9):1105-11.

6. Anders S, Pyl PT, Huber W. HTSeq--a Python framework to work with high-throughput sequencing data. Bioinformatics. 2015;31(2):166-9.

7. Robinson MD, Oshlack A. A scaling normalization method for differential expression analysis of RNA-seq data. Genome Biol. 2010;11(3):R25.

8. Chae S, Ahn BY, Byun K, Cho YM, Yu MH, Lee B, et al. A systems approach for decoding mitochondrial retrograde signaling pathways. Sci Signal. 2013;6(264):rs4.

9. Kamburov A, Wierling C, Lehrach H, Herwig R. ConsensusPathDB--a database for integrating human functional interaction networks. Nucleic Acids Res. 2009;37(Database issue):D623-8.
