# Supplementary material for: Genetic deletion of nitric oxide synthase 2 ameliorates Parkinson’s disease pathology and neuroinflammation in a transgenic mouse model of synucleinopathy
Source: Mol Brain. 2023 Jan 16;16:7. doi: 10.1186/s13041-023-00996-1 (PMC9841612; doi:10.1186/s13041-023-00996-1)
Supplement: Supplementary file 3 — Additional file 3: Table S2. One-way ANOVA (Tukey’s test) and significance of the results of the in vivo experiments in this study. [file 13041_2023_996_MOESM3_ESM.docx]

**Genetic deletion of nitric oxide synthase 2 ameliorates Parkinson’s disease pathology and neuroinflammation in a transgenic mouse model of synucleinopathy**

Jieun Kim^1,+^, Jung-Youn Han^2,+^, Yujeong Lee^3^, Kipom Kim^4^, Young Pyo Choi^2,*^, Sehyun Chae^5,*^, Hyang-Sook Hoe^1,6,*^

^1^Department of Neurodegenerative Disease, ^2^Laboratory Animal Center, ^3^Cognitive Science Research Group, ^4^Research Strategy Office, and ^5^Neurovescular Unit Research Group, Korea Brain Research Institute (KBRI), 61, Cheomdan-ro, Dong-gu, Daegu, Korea, 41062; ^6^Department of Brain and Cognitive Sciences, Daegu Gyeongbuk Institute of Science & Technology, Daegu, Korea 42988. ^+^ These authors contributed equally to this work.

^*^Corresponding author:

Hyang-Sook Hoe, Ph.D., Neurodegenerative Diseases Group, Korea Brain Research Institute (KBRI), 61, Cheomdan-ro, Dong-gu, Daegu, Korea, 41062; E-mail: [*sookhoe72@kbri.re.kr*](mailto:sookhoe72@kbri.re.kr)

Sehyun Chae, Ph.D., Neurovascular Unit Research Group, Korea Brain Research Institute (KBRI), 61, Cheomdan-ro, Dong-gu, Daegu, Korea, 41062; E-mail: *shchae@kbri.re.kr*

Young Pyo Choi, D.V.M., Ph.D., Laboratory Animal Center, Korea Brain Research Institute (KBRI), 61, Cheomdan-ro, Dong-gu, Daegu, Korea, 41062; E-mail: *cyp0201@kbri.re.kr*

**Table S2.** One-way ANOVA (Tukey’s test) and significance of the results of the in vivo experiments in this study.

| **Figure 1B: p-Syn^ser129^ fluorescence intensity-SN** |
| --- |
| \| Number of families \| 1 \|  \|  \|  \|  \|  \|  \|  \| \| --- \| --- \| --- \| --- \| --- \| --- \| --- \| --- \| --- \| \| Number of comparisons per family \| 3 \|  \|  \|  \|  \|  \|  \|  \| \| Alpha \| 0.05 \|  \|  \|  \|  \|  \|  \|  \| \|  \|  \|  \|  \|  \|  \|  \|  \|  \| \| Tukey's multiple comparisons test \| Mean Diff. \| 95.00% CI of diff. \| Significant? \| Summary \| Adjusted P Value \|  \|  \|  \| \|  \|  \|  \|  \|  \|  \|  \|  \|  \| \| Cont vs. Syn^A35T^/NOS^+/+^ \| -186 \| -253.8 to -118.1 \| Yes \| **** \| <0.0001 \| A-B \|  \|  \| \| Cont vs. SynA^35T^/NOS-^/-^ \| -51.04 \| -125.5 to 23.41 \| No \| ns \| 0.2320 \| A-C \|  \|  \| \| Syn^A35T^/NOS^+/+^ ***^vs. SynA35T/NOS-/-^*** \| 134.9 \| 62.11 to 207.7 \| Yes \| *** \| 0.0001 \| B-C \|  \|  \| \|  \|  \|  \|  \|  \|  \|  \|  \|  \| \|  \|  \|  \|  \|  \|  \|  \|  \|  \| \| Test details \| Mean 1 \| Mean 2 \| Mean Diff. \| SE of diff. \| n1 \| n2 \| q \| DF \| \|  \|  \|  \|  \|  \|  \|  \|  \|  \| \| Cont vs. Syn^A35T^/NOS^+/+^ \| 100 \| 286 \| -186 \| 28.08 \| 18 \| 20 \| 9.364 \| 49 \| \| Cont vs. SynA^35T^/NOS-^/-^ \| 100 \| 151 \| -51.04 \| 30.8 \| 18 \| 14 \| 2.343 \| 49 \| \| Syn^A35T^/NOS^+/+^ ***^vs. SynA35T/NOS-/-^*** \| 286 \| 151 \| 134.9 \| 30.12 \| 20 \| 14 \| 6.334 \| 49 \| |
| **Figure 1B: p-Syn^ser129^ fluorescence intensity-DpMe** |
| \| Number of families \| 1 \|  \|  \|  \|  \|  \|  \|  \| \| --- \| --- \| --- \| --- \| --- \| --- \| --- \| --- \| --- \| \| Number of comparisons per family \| 3 \|  \|  \|  \|  \|  \|  \|  \| \| Alpha \| 0.05 \|  \|  \|  \|  \|  \|  \|  \| \|  \|  \|  \|  \|  \|  \|  \|  \|  \| \| Tukey's multiple comparisons test \| Mean Diff. \| 95.00% CI of diff. \| Significant? \| Summary \| Adjusted P Value \|  \|  \|  \| \|  \|  \|  \|  \|  \|  \|  \|  \|  \| \| Cont vs. Syn^A35T^/NOS^+/+^ \| -222.5 \| -360.5 to -84.42 \| Yes \| *** \| 0.0009 \| D-E \|  \|  \| \| Cont vs. SynA^35T^/NOS-^/-^ \| -8.959 \| -156.9 to 139 \| No \| ns \| 0.9882 \| D-F \|  \|  \| \| Syn^A35T^/NOS^+/+^ ***^vs. SynA35T/NOS-/-^*** \| 213.5 \| 72.93 to 354.1 \| Yes \| ** \| 0.0017 \| E-F \|  \|  \| \|  \|  \|  \|  \|  \|  \|  \|  \|  \| \|  \|  \|  \|  \|  \|  \|  \|  \|  \| \| Test details \| Mean 1 \| Mean 2 \| Mean Diff. \| SE of diff. \| n1 \| n2 \| q \| DF \| \|  \|  \|  \|  \|  \|  \|  \|  \|  \| \| Cont vs. Syn^A35T^/NOS^+/+^ \| 100 \| 322.5 \| -222.5 \| 57.08 \| 16 \| 20 \| 5.512 \| 48 \| \| Cont vs. SynA^35T^/NOS-^/-^ \| 100 \| 109 \| -8.959 \| 61.16 \| 16 \| 15 \| 0.2072 \| 48 \| \| Syn^A35T^/NOS^+/+^ ***^vs. SynA35T/NOS-/-^*** \| 322.5 \| 109 \| 213.5 \| 58.13 \| 20 \| 15 \| 5.195 \| 48 \| |
| **Figure 1B: p-Syn^ser129^ fluorescence intensity-Gi** |
| \| Number of families \| 1 \|  \|  \|  \|  \|  \|  \|  \| \| --- \| --- \| --- \| --- \| --- \| --- \| --- \| --- \| --- \| \| Number of comparisons per family \| 3 \|  \|  \|  \|  \|  \|  \|  \| \| Alpha \| 0.05 \|  \|  \|  \|  \|  \|  \|  \| \|  \|  \|  \|  \|  \|  \|  \|  \|  \| \| Tukey's multiple comparisons test \| Mean Diff. \| 95.00% CI of diff. \| Significant? \| Summary \| Adjusted P Value \|  \|  \|  \| \|  \|  \|  \|  \|  \|  \|  \|  \|  \| \| Cont vs. Syn^A35T^/NOS^+/+^ \| -267.7 \| -350.5 to -185 \| Yes \| **** \| <0.0001 \| G-H \|  \|  \| \| Cont vs. SynA^35T^/NOS-^/-^ \| -65.27 \| -155.1 to 24.55 \| No \| ns \| 0.1960 \| G-I \|  \|  \| \| Syn^A35T^/NOS^+/+^ vs. SynA^35T^/NOS-^/-^ \| 202.5 \| 114.5 to 290.5 \| Yes \| **** \| <0.0001 \| H-I \|  \|  \| \|  \|  \|  \|  \|  \|  \|  \|  \|  \| \|  \|  \|  \|  \|  \|  \|  \|  \|  \| \| Test details \| Mean 1 \| Mean 2 \| Mean Diff. \| SE of diff. \| n1 \| n2 \| q \| DF \| \|  \|  \|  \|  \|  \|  \|  \|  \|  \| \| Cont vs. Syn^A35T^/NOS^+/+^ \| 100 \| 367.7 \| -267.7 \| 34.35 \| 20 \| 22 \| 11.02 \| 55 \| \| Cont vs. SynA^35T^/NOS-^/-^ \| 100 \| 165.3 \| -65.27 \| 37.29 \| 20 \| 16 \| 2.475 \| 55 \| \| Syn^A35T^/NOS^+/+^ vs. SynA^35T^/NOS-^/-^ \| 367.7 \| 165.3 \| 202.5 \| 36.53 \| 22 \| 16 \| 7.839 \| 55 \| |
| **Figure 1D: Iba-1 fluorescence intensity-SN** |
| \| Number of families \| 1 \|  \|  \|  \|  \|  \|  \|  \| \| --- \| --- \| --- \| --- \| --- \| --- \| --- \| --- \| --- \| \| Number of comparisons per family \| 3 \|  \|  \|  \|  \|  \|  \|  \| \| Alpha \| 0.05 \|  \|  \|  \|  \|  \|  \|  \| \|  \|  \|  \|  \|  \|  \|  \|  \|  \| \| Tukey's multiple comparisons test \| Mean Diff. \| 95.00% CI of diff. \| Significant? \| Summary \| Adjusted P Value \|  \|  \|  \| \|  \|  \|  \|  \|  \|  \|  \|  \|  \| \| Cont vs. Syn^A35T^/NOS^+/+^ \| 30.25 \| 2.604 to 57.91 \| Yes \| * \| 0.0290 \| A-B \|  \|  \| \| Cont vs. SynA^35T^/NOS-^/-^ \| 62.47 \| 33.27 to 91.68 \| Yes \| **** \| <0.0001 \| A-C \|  \|  \| \| Syn^A35T^/NOS^+/+^ vs. SynA^35T^/NOS-^/-^ \| 32.22 \| 1.968 to 62.47 \| Yes \| * \| 0.0345 \| B-C \|  \|  \| \|  \|  \|  \|  \|  \|  \|  \|  \|  \| \|  \|  \|  \|  \|  \|  \|  \|  \|  \| \| Test details \| Mean 1 \| Mean 2 \| Mean Diff. \| SE of diff. \| n1 \| n2 \| q \| DF \| \|  \|  \|  \|  \|  \|  \|  \|  \|  \| \| Cont vs. Syn^A35T^/NOS^+/+^ \| 100 \| 69.75 \| 30.25 \| 11.43 \| 20 \| 17 \| 3.742 \| 48 \| \| Cont vs. SynA^35T^/NOS-^/-^ \| 100 \| 37.53 \| 62.47 \| 12.08 \| 20 \| 14 \| 7.316 \| 48 \| \| Syn^A35T^/NOS^+/+^ vs. SynA^35T^/NOS-^/-^ \| 69.75 \| 37.53 \| 32.22 \| 12.51 \| 17 \| 14 \| 3.643 \| 48 \| |
| **Figure 1D: Iba-1 fluorescence intensity-DpMe** |
| \| Number of families \| 1 \|  \|  \|  \|  \|  \|  \|  \| \| --- \| --- \| --- \| --- \| --- \| --- \| --- \| --- \| --- \| \| Number of comparisons per family \| 3 \|  \|  \|  \|  \|  \|  \|  \| \| Alpha \| 0.05 \|  \|  \|  \|  \|  \|  \|  \| \|  \|  \|  \|  \|  \|  \|  \|  \|  \| \| Tukey's multiple comparisons test \| Mean Diff. \| 95.00% CI of diff. \| Significant? \| Summary \| Adjusted P Value \|  \|  \|  \| \|  \|  \|  \|  \|  \|  \|  \|  \|  \| \| Cont vs. Syn^A35T^/NOS^+/+^ \| -9.941 \| -47.66 to 27.78 \| No \| ns \| 0.8006 \| D-E \|  \|  \| \| Cont vs. SynA^35T^/NOS-^/-^ \| 99.46 \| 60.4 to 138.5 \| Yes \| **** \| <0.0001 \| D-F \|  \|  \| \| Syn^A35T^/NOS^+/+^ vs. SynA^35T^/NOS-^/-^ \| 109.4 \| 68.89 to 149.9 \| Yes \| **** \| <0.0001 \| E-F \|  \|  \| \|  \|  \|  \|  \|  \|  \|  \|  \|  \| \|  \|  \|  \|  \|  \|  \|  \|  \|  \| \| Test details \| Mean 1 \| Mean 2 \| Mean Diff. \| SE of diff. \| n1 \| n2 \| q \| DF \| \|  \|  \|  \|  \|  \|  \|  \|  \|  \| \| Cont vs. Syn^A35T^/NOS^+/+^ \| 100 \| 109.9 \| -9.941 \| 15.61 \| 20 \| 17 \| 0.9007 \| 49 \| \| Cont vs. SynA^35T^/NOS-^/-^ \| 100 \| 0.5429 \| 99.46 \| 16.16 \| 20 \| 15 \| 8.704 \| 49 \| \| Syn^A35T^/NOS^+/+^ vs. SynA^35T^/NOS-^/-^ \| 109.9 \| 0.5429 \| 109.4 \| 16.76 \| 17 \| 15 \| 9.231 \| 49 \| |
| **Figure 1D: Iba-1 fluorescence intensity-Gi** |
| \| Number of families \| 1 \|  \|  \|  \|  \|  \|  \|  \| \| --- \| --- \| --- \| --- \| --- \| --- \| --- \| --- \| --- \| \| Number of comparisons per family \| 3 \|  \|  \|  \|  \|  \|  \|  \| \| Alpha \| 0.05 \|  \|  \|  \|  \|  \|  \|  \| \|  \|  \|  \|  \|  \|  \|  \|  \|  \| \| Tukey's multiple comparisons test \| Mean Diff. \| 95.00% CI of diff. \| Significant? \| Summary \| Adjusted P Value \|  \|  \|  \| \|  \|  \|  \|  \|  \|  \|  \|  \|  \| \| Cont vs. Syn^A35T^/NOS^+/+^ \| -47.82 \| -95.07 to -0.5727 \| Yes \| * \| 0.0467 \| G-H \|  \|  \| \| Cont vs. SynA^35T^/NOS-^/-^ \| 99.42 \| 43.33 to 155.5 \| Yes \| *** \| 0.0003 \| G-I \|  \|  \| \| Syn^A35T^/NOS^+/+^ vs. SynA^35T^/NOS-^/-^ \| 147.2 \| 91.15 to 203.3 \| Yes \| **** \| <0.0001 \| H-I \|  \|  \| \|  \|  \|  \|  \|  \|  \|  \|  \|  \| \|  \|  \|  \|  \|  \|  \|  \|  \|  \| \| Test details \| Mean 1 \| Mean 2 \| Mean Diff. \| SE of diff. \| n1 \| n2 \| q \| DF \| \|  \|  \|  \|  \|  \|  \|  \|  \|  \| \| Cont vs. Syn^A35T^/NOS^+/+^ \| 100 \| 147.8 \| -47.82 \| 19.54 \| 20 \| 20 \| 3.462 \| 48 \| \| Cont vs. SynA^35T^/NOS-^/-^ \| 100 \| 0.5824 \| 99.42 \| 23.19 \| 20 \| 11 \| 6.063 \| 48 \| \| Syn^A35T^/NOS^+/+^ vs. SynA^35T^/NOS-^/-^ \| 147.8 \| 0.5824 \| 147.2 \| 23.19 \| 20 \| 11 \| 8.979 \| 48 \| |
| **Figure 1F: GFAP fluorescence intensity-SN** |
| \| Number of families \| 1 \|  \|  \|  \|  \|  \|  \|  \| \| --- \| --- \| --- \| --- \| --- \| --- \| --- \| --- \| --- \| \| Number of comparisons per family \| 3 \|  \|  \|  \|  \|  \|  \|  \| \| Alpha \| 0.05 \|  \|  \|  \|  \|  \|  \|  \| \|  \|  \|  \|  \|  \|  \|  \|  \|  \| \| Tukey's multiple comparisons test \| Mean Diff. \| 95.00% CI of diff. \| Significant? \| Summary \| Adjusted P Value \|  \|  \|  \| \|  \|  \|  \|  \|  \|  \|  \|  \|  \| \| Cont vs. Syn^A35T^/NOS^+/+^ \| 11.19 \| -17.87 to 40.26 \| No \| ns \| 0.6233 \| A-B \|  \|  \| \| Cont vs. SynA^35T^/NOS-^/-^ \| 46.86 \| 16.15 to 77.56 \| Yes \| ** \| 0.0016 \| A-C \|  \|  \| \| Syn^A35T^/NOS^+/+^ vs. SynA^35T^/NOS-^/-^ \| 35.66 \| 3.862 to 67.47 \| Yes \| * \| 0.0246 \| B-C \|  \|  \| \|  \|  \|  \|  \|  \|  \|  \|  \|  \| \|  \|  \|  \|  \|  \|  \|  \|  \|  \| \| Test details \| Mean 1 \| Mean 2 \| Mean Diff. \| SE of diff. \| n1 \| n2 \| q \| DF \| \|  \|  \|  \|  \|  \|  \|  \|  \|  \| \| Cont vs. Syn^A35T^/NOS^+/+^ \| 100 \| 88.81 \| 11.19 \| 12.02 \| 20 \| 17 \| 1.317 \| 48 \| \| Cont vs. SynA^35T^/NOS-^/-^ \| 100 \| 53.14 \| 46.86 \| 12.7 \| 20 \| 14 \| 5.219 \| 48 \| \| Syn^A35T^/NOS^+/+^ vs. SynA^35T^/NOS-^/-^ \| 88.81 \| 53.14 \| 35.66 \| 13.15 \| 17 \| 14 \| 3.836 \| 48 \| |
| **Figure 1F: GFAP fluorescence intensity-DpMe** |
| \| Number of families \| 1 \|  \|  \|  \|  \|  \|  \|  \| \| --- \| --- \| --- \| --- \| --- \| --- \| --- \| --- \| --- \| \| Number of comparisons per family \| 3 \|  \|  \|  \|  \|  \|  \|  \| \| Alpha \| 0.05 \|  \|  \|  \|  \|  \|  \|  \| \|  \|  \|  \|  \|  \|  \|  \|  \|  \| \| Tukey's multiple comparisons test \| Mean Diff. \| 95.00% CI of diff. \| Significant? \| Summary \| Adjusted P Value \|  \|  \|  \| \|  \|  \|  \|  \|  \|  \|  \|  \|  \| \| Cont vs. Syn^A35T^/NOS^+/+^ \| -16.88 \| -46.37 to 12.61 \| No \| ns \| 0.3576 \| D-E \|  \|  \| \| Cont vs. SynA^35T^/NOS-^/-^ \| 62.4 \| 32.42 to 92.39 \| Yes \| **** \| <0.0001 \| D-F \|  \|  \| \| Syn^A35T^/NOS^+/+^ vs. SynA^35T^/NOS-^/-^ \| 79.28 \| 48.14 to 110.4 \| Yes \| **** \| <0.0001 \| E-F \|  \|  \| \|  \|  \|  \|  \|  \|  \|  \|  \|  \| \|  \|  \|  \|  \|  \|  \|  \|  \|  \| \| Test details \| Mean 1 \| Mean 2 \| Mean Diff. \| SE of diff. \| n1 \| n2 \| q \| DF \| \|  \|  \|  \|  \|  \|  \|  \|  \|  \| \| Cont vs. Syn^A35T^/NOS^+/+^ \| 100 \| 116.9 \| -16.88 \| 12.21 \| 20 \| 17 \| 1.955 \| 50 \| \| Cont vs. SynA^35T^/NOS-^/-^ \| 100 \| 37.6 \| 62.4 \| 12.41 \| 20 \| 16 \| 7.109 \| 50 \| \| Syn^A35T^/NOS^+/+^ vs. SynA^35T^/NOS-^/-^ \| 116.9 \| 37.6 \| 79.28 \| 12.89 \| 17 \| 16 \| 8.697 \| 50 \| |
| **Figure 1F: GFAP fluorescence intensity-Gi** |
| \| Number of families \| 1 \|  \|  \|  \|  \|  \|  \|  \| \| --- \| --- \| --- \| --- \| --- \| --- \| --- \| --- \| --- \| \| Number of comparisons per family \| 3 \|  \|  \|  \|  \|  \|  \|  \| \| Alpha \| 0.05 \|  \|  \|  \|  \|  \|  \|  \| \|  \|  \|  \|  \|  \|  \|  \|  \|  \| \| Tukey's multiple comparisons test \| Mean Diff. \| 95.00% CI of diff. \| Significant? \| Summary \| Adjusted P Value \|  \|  \|  \| \|  \|  \|  \|  \|  \|  \|  \|  \|  \| \| Cont vs. Syn^A35T^/NOS^+/+^ \| -37.38 \| -74.09 to -0.6603 \| Yes \| * \| 0.0452 \| G-H \|  \|  \| \| Cont vs. SynA^35T^/NOS-^/-^ \| 67.68 \| 25.28 to 110.1 \| Yes \| *** \| 0.0010 \| G-I \|  \|  \| \| Syn^A35T^/NOS^+/+^ vs. SynA^35T^/NOS-^/-^ \| 105.1 \| 62.66 to 147.5 \| Yes \| **** \| <0.0001 \| H-I \|  \|  \| \|  \|  \|  \|  \|  \|  \|  \|  \|  \| \|  \|  \|  \|  \|  \|  \|  \|  \|  \| \| Test details \| Mean 1 \| Mean 2 \| Mean Diff. \| SE of diff. \| n1 \| n2 \| q \| DF \| \|  \|  \|  \|  \|  \|  \|  \|  \|  \| \| Cont vs. Syn^A35T^/NOS^+/+^ \| 100 \| 137.4 \| -37.38 \| 15.19 \| 20 \| 20 \| 3.48 \| 49 \| \| Cont vs. SynA^35T^/NOS-^/-^ \| 100 \| 32.32 \| 67.68 \| 17.54 \| 20 \| 12 \| 5.456 \| 49 \| \| Syn^A35T^/NOS^+/+^ vs. SynA^35T^/NOS-^/-^ \| 137.4 \| 32.32 \| 105.1 \| 17.54 \| 20 \| 12 \| 8.47 \| 49 \| |
| **Figure S2B: p-Syn^ser129^ fluorescence intensity-Cortex** |
| \| Number of families \| 1 \|  \|  \|  \|  \|  \|  \|  \| \| --- \| --- \| --- \| --- \| --- \| --- \| --- \| --- \| --- \| \| Number of comparisons per family \| 3 \|  \|  \|  \|  \|  \|  \|  \| \| Alpha \| 0.05 \|  \|  \|  \|  \|  \|  \|  \| \|  \|  \|  \|  \|  \|  \|  \|  \|  \| \| Tukey's multiple comparisons test \| Mean Diff. \| 95.00% CI of diff. \| Significant? \| Summary \| Adjusted P Value \|  \|  \|  \| \|  \|  \|  \|  \|  \|  \|  \|  \|  \| \| nTg vs. Syn^A35T^ \| -230 \| -303.7 to -156.4 \| Yes \| **** \| <0.0001 \| A-B \|  \|  \| \| nTg vs. Syn^A35T^ NOS2^-/-^ \| -52.3 \| -132.9 to 28.29 \| No \| ns \| 0.2691 \| A-C \|  \|  \| \| Syn^A35T^ vs. Syn^A35T^ NOS2^-/-^ \| 177.7 \| 102.7 to 252.8 \| Yes \| **** \| <0.0001 \| B-C \|  \|  \| \|  \|  \|  \|  \|  \|  \|  \|  \|  \| \|  \|  \|  \|  \|  \|  \|  \|  \|  \| \| Test details \| Mean 1 \| Mean 2 \| Mean Diff. \| SE of diff. \| n1 \| n2 \| q \| DF \| \|  \|  \|  \|  \|  \|  \|  \|  \|  \| \| nTg vs. Syn^A35T^ \| 100 \| 330 \| -230 \| 30.5 \| 16 \| 22 \| 10.67 \| 50 \| \| nTg vs. Syn^A35T^ NOS2^-/-^ \| 100 \| 152.3 \| -52.3 \| 33.36 \| 16 \| 15 \| 2.217 \| 50 \| \| Syn^A35T^ vs. Syn^A35T^ NOS2^-/-^ \| 330 \| 152.3 \| 177.7 \| 31.08 \| 22 \| 15 \| 8.087 \| 50 \| |
| **Figure S2B: p-Syn^ser129^ fluorescence intensity-CPu** |
| \| Number of families \| 1 \|  \|  \|  \|  \|  \|  \|  \| \| --- \| --- \| --- \| --- \| --- \| --- \| --- \| --- \| --- \| \| Number of comparisons per family \| 3 \|  \|  \|  \|  \|  \|  \|  \| \| Alpha \| 0.05 \|  \|  \|  \|  \|  \|  \|  \| \|  \|  \|  \|  \|  \|  \|  \|  \|  \| \| Tukey's multiple comparisons test \| Mean Diff. \| 95.00% CI of diff. \| Significant? \| Summary \| Adjusted P Value \|  \|  \|  \| \|  \|  \|  \|  \|  \|  \|  \|  \|  \| \| nTg vs. Syn^A35T^ \| -168.7 \| -213.1 to -124.4 \| Yes \| **** \| <0.0001 \| D-E \|  \|  \| \| nTg vs. Syn^A35T^ NOS2^-/-^ \| 56.35 \| 7.333 to 105.4 \| Yes \| * \| 0.0206 \| D-F \|  \|  \| \| Syn^A35T^ vs. Syn^A35T^ NOS2^-/-^ \| 225.1 \| 177 to 273.1 \| Yes \| **** \| <0.0001 \| E-F \|  \|  \| \|  \|  \|  \|  \|  \|  \|  \|  \|  \| \|  \|  \|  \|  \|  \|  \|  \|  \|  \| \| Test details \| Mean 1 \| Mean 2 \| Mean Diff. \| SE of diff. \| n1 \| n2 \| q \| DF \| \|  \|  \|  \|  \|  \|  \|  \|  \|  \| \| nTg vs. Syn^A35T^ \| 100 \| 268.7 \| -168.7 \| 18.4 \| 20 \| 22 \| 12.97 \| 54 \| \| nTg vs. Syn^A35T^ NOS2^-/-^ \| 100 \| 43.65 \| 56.35 \| 20.34 \| 20 \| 15 \| 3.918 \| 54 \| \| Syn^A35T^ vs. Syn^A35T^ NOS2^-/-^ \| 268.7 \| 43.65 \| 225.1 \| 19.94 \| 22 \| 15 \| 15.96 \| 54 \| |
| **Figure S2B: p-Syn^ser129^ fluorescence intensity-Hippo** |
| \| Number of families \| 1 \|  \|  \|  \|  \|  \|  \|  \| \| --- \| --- \| --- \| --- \| --- \| --- \| --- \| --- \| --- \| \| Number of comparisons per family \| 3 \|  \|  \|  \|  \|  \|  \|  \| \| Alpha \| 0.05 \|  \|  \|  \|  \|  \|  \|  \| \|  \|  \|  \|  \|  \|  \|  \|  \|  \| \| Tukey's multiple comparisons test \| Mean Diff. \| 95.00% CI of diff. \| Significant? \| Summary \| Adjusted P Value \|  \|  \|  \| \|  \|  \|  \|  \|  \|  \|  \|  \|  \| \| nTg vs. Syn^A35T^ \| -384.3 \| -479.8 to -288.8 \| Yes \| **** \| <0.0001 \| G-H \|  \|  \| \| nTg vs. Syn^A35T^ NOS2^-/-^ \| -51.23 \| -159 to 56.51 \| No \| ns \| 0.4896 \| G-I \|  \|  \| \| Syn^A35T^ vs. Syn^A35T^ NOS2^-/-^ \| 333 \| 231.9 to 434.2 \| Yes \| **** \| <0.0001 \| H-I \|  \|  \| \|  \|  \|  \|  \|  \|  \|  \|  \|  \| \|  \|  \|  \|  \|  \|  \|  \|  \|  \| \| Test details \| Mean 1 \| Mean 2 \| Mean Diff. \| SE of diff. \| n1 \| n2 \| q \| DF \| \|  \|  \|  \|  \|  \|  \|  \|  \|  \| \| nTg vs. Syn^A35T^ \| 100 \| 484.3 \| -384.3 \| 39.55 \| 17 \| 23 \| 13.74 \| 51 \| \| nTg vs. Syn^A35T^ NOS2^-/-^ \| 100 \| 151.2 \| -51.23 \| 44.63 \| 17 \| 14 \| 1.623 \| 51 \| \| Syn^A35T^ vs. Syn^A35T^ NOS2^-/-^ \| 484.3 \| 151.2 \| 333 \| 41.92 \| 23 \| 14 \| 11.24 \| 51 \| |
| **Figure S3A: Iba-1 cells-SN** |
| \| Number of families \| 1 \|  \|  \|  \|  \|  \|  \|  \| \| --- \| --- \| --- \| --- \| --- \| --- \| --- \| --- \| --- \| \| Number of comparisons per family \| 3 \|  \|  \|  \|  \|  \|  \|  \| \| Alpha \| 0.05 \|  \|  \|  \|  \|  \|  \|  \| \|  \|  \|  \|  \|  \|  \|  \|  \|  \| \| Tukey's multiple comparisons test \| Mean Diff. \| 95.00% CI of diff. \| Significant? \| Summary \| Adjusted P Value \|  \|  \|  \| \|  \|  \|  \|  \|  \|  \|  \|  \|  \| \| Cont vs. Syn^A35T^/NOS^+/+^ \| 3596222 \| -4440190 to 11632635 \| No \| ns \| 0.5296 \| A-B \|  \|  \| \| Cont vs. SynA^35T^/NOS-^/-^ \| 12841004 \| 4351912 to 21330096 \| Yes \| ** \| 0.0018 \| A-C \|  \|  \| \| Syn^A35T^/NOS^+/+^ vs. SynA^35T^/NOS-^/-^ \| 9244782 \| 452670 to 18036893 \| Yes \| * \| 0.0373 \| B-C \|  \|  \| \|  \|  \|  \|  \|  \|  \|  \|  \|  \| \|  \|  \|  \|  \|  \|  \|  \|  \|  \| \| Test details \| Mean 1 \| Mean 2 \| Mean Diff. \| SE of diff. \| n1 \| n2 \| q \| DF \| \|  \|  \|  \|  \|  \|  \|  \|  \|  \| \| Cont vs. Syn^A35T^/NOS^+/+^ \| 20494494 \| 16898272 \| 3596222 \| 3322908 \| 20 \| 17 \| 1.531 \| 48 \| \| Cont vs. SynA^35T^/NOS-^/-^ \| 20494494 \| 7653490 \| 12841004 \| 3510083 \| 20 \| 14 \| 5.174 \| 48 \| \| Syn^A35T^/NOS^+/+^ vs. SynA^35T^/NOS-^/-^ \| 16898272 \| 7653490 \| 9244782 \| 3635376 \| 17 \| 14 \| 3.596 \| 48 \| |
| **Figure S3A: Iba-1 cells-DpMe** |
| \| Number of families \| 1 \|  \|  \|  \|  \|  \|  \|  \| \| --- \| --- \| --- \| --- \| --- \| --- \| --- \| --- \| --- \| \| Number of comparisons per family \| 3 \|  \|  \|  \|  \|  \|  \|  \| \| Alpha \| 0.05 \|  \|  \|  \|  \|  \|  \|  \| \|  \|  \|  \|  \|  \|  \|  \|  \|  \| \| Tukey's multiple comparisons test \| Mean Diff. \| 95.00% CI of diff. \| Significant? \| Summary \| Adjusted P Value \|  \|  \|  \| \|  \|  \|  \|  \|  \|  \|  \|  \|  \| \| Cont vs. Syn^A35T^/NOS^+/+^ \| -8481509 \| -17842711 to 879692 \| No \| ns \| 0.0830 \| D-E \|  \|  \| \| Cont vs. SynA^35T^/NOS-^/-^ \| 8380654 \| -1137363 to 17898671 \| No \| ns \| 0.0946 \| D-F \|  \|  \| \| Syn^A35T^/NOS^+/+^ vs. SynA^35T^/NOS-^/-^ \| 16862163 \| 6977936 to 26746390 \| Yes \| *** \| 0.0004 \| E-F \|  \|  \| \|  \|  \|  \|  \|  \|  \|  \|  \|  \| \|  \|  \|  \|  \|  \|  \|  \|  \|  \| \| Test details \| Mean 1 \| Mean 2 \| Mean Diff. \| SE of diff. \| n1 \| n2 \| q \| DF \| \|  \|  \|  \|  \|  \|  \|  \|  \|  \| \| Cont vs. Syn^A35T^/NOS^+/+^ \| 8820685 \| 17302194 \| -8481509 \| 3875599 \| 20 \| 17 \| 3.095 \| 50 \| \| Cont vs. SynA^35T^/NOS-^/-^ \| 8820685 \| 440031 \| 8380654 \| 3940521 \| 20 \| 16 \| 3.008 \| 50 \| \| Syn^A35T^/NOS^+/+^ vs. SynA^35T^/NOS-^/-^ \| 17302194 \| 440031 \| 16862163 \| 4092135 \| 17 \| 16 \| 5.827 \| 50 \| |
| **Figure S3A: Iba-1 cells-Gi** |
| \| Number of families \| 1 \|  \|  \|  \|  \|  \|  \|  \| \| --- \| --- \| --- \| --- \| --- \| --- \| --- \| --- \| --- \| \| Number of comparisons per family \| 3 \|  \|  \|  \|  \|  \|  \|  \| \| Alpha \| 0.05 \|  \|  \|  \|  \|  \|  \|  \| \|  \|  \|  \|  \|  \|  \|  \|  \|  \| \| Tukey's multiple comparisons test \| Mean Diff. \| 95.00% CI of diff. \| Significant? \| Summary \| Adjusted P Value \|  \|  \|  \| \|  \|  \|  \|  \|  \|  \|  \|  \|  \| \| Cont vs. Syn^A35T^/NOS^+/+^ \| -11592269 \| -20131736 to -3052802 \| Yes \| ** \| 0.0054 \| G-H \|  \|  \| \| Cont vs. SynA^35T^/NOS-^/-^ \| 8090557 \| -2046229 to 18227342 \| No \| ns \| 0.1412 \| G-I \|  \|  \| \| Syn^A35T^/NOS^+/+^ vs. SynA^35T^/NOS-^/-^ \| 19682826 \| 9546040 to 29819611 \| Yes \| **** \| <0.0001 \| H-I \|  \|  \| \|  \|  \|  \|  \|  \|  \|  \|  \|  \| \|  \|  \|  \|  \|  \|  \|  \|  \|  \| \| Test details \| Mean 1 \| Mean 2 \| Mean Diff. \| SE of diff. \| n1 \| n2 \| q \| DF \| \|  \|  \|  \|  \|  \|  \|  \|  \|  \| \| Cont vs. Syn^A35T^/NOS^+/+^ \| 8633447 \| 20225716 \| -11592269 \| 3530912 \| 20 \| 20 \| 4.643 \| 48 \| \| Cont vs. SynA^35T^/NOS-^/-^ \| 8633447 \| 542891 \| 8090557 \| 4191374 \| 20 \| 11 \| 2.73 \| 48 \| \| Syn^A35T^/NOS^+/+^ vs. SynA^35T^/NOS-^/-^ \| 20225716 \| 542891 \| 19682826 \| 4191374 \| 20 \| 11 \| 6.641 \| 48 \| |
| **Figure S3B: Iba-1 % area-SN** |
| \| Number of families \| 1 \|  \|  \|  \|  \|  \|  \|  \| \| --- \| --- \| --- \| --- \| --- \| --- \| --- \| --- \| --- \| \| Number of comparisons per family \| 3 \|  \|  \|  \|  \|  \|  \|  \| \| Alpha \| 0.05 \|  \|  \|  \|  \|  \|  \|  \| \|  \|  \|  \|  \|  \|  \|  \|  \|  \| \| Tukey's multiple comparisons test \| Mean Diff. \| 95.00% CI of diff. \| Significant? \| Summary \| Adjusted P Value \|  \|  \|  \| \|  \|  \|  \|  \|  \|  \|  \|  \|  \| \| Cont vs. Syn^A35T^/NOS^+/+^ \| 1.176 \| -1.363 to 3.714 \| No \| ns \| 0.5066 \| A-B \|  \|  \| \| Cont vs. SynA^35T^/NOS-^/-^ \| 4.165 \| 1.484 to 6.847 \| Yes \| ** \| 0.0013 \| A-C \|  \|  \| \| Syn^A35T^/NOS^+/+^ vs. SynA^35T^/NOS-^/-^ \| 2.99 \| 0.2124 to 5.767 \| Yes \| * \| 0.0322 \| B-C \|  \|  \| \|  \|  \|  \|  \|  \|  \|  \|  \|  \| \|  \|  \|  \|  \|  \|  \|  \|  \|  \| \| Test details \| Mean 1 \| Mean 2 \| Mean Diff. \| SE of diff. \| n1 \| n2 \| q \| DF \| \|  \|  \|  \|  \|  \|  \|  \|  \|  \| \| Cont vs. Syn^A35T^/NOS^+/+^ \| 5.443 \| 4.268 \| 1.176 \| 1.05 \| 20 \| 17 \| 1.584 \| 48 \| \| Cont vs. SynA^35T^/NOS-^/-^ \| 5.443 \| 1.278 \| 4.165 \| 1.109 \| 20 \| 14 \| 5.313 \| 48 \| \| Syn^A35T^/NOS^+/+^ vs. SynA^35T^/NOS-^/-^ \| 4.268 \| 1.278 \| 2.99 \| 1.148 \| 17 \| 14 \| 3.682 \| 48 \| |
| **Figure S3B: Iba-1 % area-DpMe** |
| \| Number of families \| 1 \|  \|  \|  \|  \|  \|  \|  \| \| --- \| --- \| --- \| --- \| --- \| --- \| --- \| --- \| --- \| \| Number of comparisons per family \| 3 \|  \|  \|  \|  \|  \|  \|  \| \| Alpha \| 0.05 \|  \|  \|  \|  \|  \|  \|  \| \|  \|  \|  \|  \|  \|  \|  \|  \|  \| \| Tukey's multiple comparisons test \| Mean Diff. \| 95.00% CI of diff. \| Significant? \| Summary \| Adjusted P Value \|  \|  \|  \| \|  \|  \|  \|  \|  \|  \|  \|  \|  \| \| Cont vs. Syn^A35T^/NOS^+/+^ \| -3.537 \| -5.882 to -1.192 \| Yes \| ** \| 0.0018 \| D-E \|  \|  \| \| Cont vs. SynA^35T^/NOS-^/-^ \| 1.406 \| -0.9779 to 3.791 \| No \| ns \| 0.3361 \| D-F \|  \|  \| \| Syn^A35T^/NOS^+/+^ vs. SynA^35T^/NOS-^/-^ \| 4.944 \| 2.468 to 7.42 \| Yes \| **** \| <0.0001 \| E-F \|  \|  \| \|  \|  \|  \|  \|  \|  \|  \|  \|  \| \|  \|  \|  \|  \|  \|  \|  \|  \|  \| \| Test details \| Mean 1 \| Mean 2 \| Mean Diff. \| SE of diff. \| n1 \| n2 \| q \| DF \| \|  \|  \|  \|  \|  \|  \|  \|  \|  \| \| Cont vs. Syn^A35T^/NOS^+/+^ \| 1.42 \| 4.957 \| -3.537 \| 0.9709 \| 20 \| 17 \| 5.153 \| 50 \| \| Cont vs. SynA^35T^/NOS-^/-^ \| 1.42 \| 0.01318 \| 1.406 \| 0.9872 \| 20 \| 16 \| 2.015 \| 50 \| \| Syn^A35T^/NOS^+/+^ vs. SynA^35T^/NOS-^/-^ \| 4.957 \| 0.01318 \| 4.944 \| 1.025 \| 17 \| 16 \| 6.82 \| 50 \| |
| **Figure S3B: Iba-1 % area-Gi** |
| \| Number of families \| 1 \|  \|  \|  \|  \|  \|  \|  \| \| --- \| --- \| --- \| --- \| --- \| --- \| --- \| --- \| --- \| \| Number of comparisons per family \| 3 \|  \|  \|  \|  \|  \|  \|  \| \| Alpha \| 0.05 \|  \|  \|  \|  \|  \|  \|  \| \|  \|  \|  \|  \|  \|  \|  \|  \|  \| \| Tukey's multiple comparisons test \| Mean Diff. \| 95.00% CI of diff. \| Significant? \| Summary \| Adjusted P Value \|  \|  \|  \| \|  \|  \|  \|  \|  \|  \|  \|  \|  \| \| Cont vs. Syn^A35T^/NOS^+/+^ \| -11.93 \| -19.86 to -4.007 \| Yes \| ** \| 0.0019 \| G-H \|  \|  \| \| Cont vs. SynA^35T^/NOS-^/-^ \| 1.472 \| -7.935 to 10.88 \| No \| ns \| 0.9242 \| G-I \|  \|  \| \| Syn^A35T^/NOS^+/+^ vs. SynA^35T^/NOS-^/-^ \| 13.4 \| 3.997 to 22.81 \| Yes \| ** \| 0.0034 \| H-I \|  \|  \| \|  \|  \|  \|  \|  \|  \|  \|  \|  \| \|  \|  \|  \|  \|  \|  \|  \|  \|  \| \| Test details \| Mean 1 \| Mean 2 \| Mean Diff. \| SE of diff. \| n1 \| n2 \| q \| DF \| \|  \|  \|  \|  \|  \|  \|  \|  \|  \| \| Cont vs. Syn^A35T^/NOS^+/+^ \| 1.486 \| 13.42 \| -11.93 \| 3.276 \| 20 \| 20 \| 5.15 \| 48 \| \| Cont vs. SynA^35T^/NOS-^/-^ \| 1.486 \| 0.01445 \| 1.472 \| 3.889 \| 20 \| 11 \| 0.5351 \| 48 \| \| Syn^A35T^/NOS^+/+^ vs. SynA^35T^/NOS-^/-^ \| 13.42 \| 0.01445 \| 13.4 \| 3.889 \| 20 \| 11 \| 4.874 \| 48 \| |
| **Figure S3C: GFAP cells-SN** |
| \| Number of families \| 1 \|  \|  \|  \|  \|  \|  \|  \| \| --- \| --- \| --- \| --- \| --- \| --- \| --- \| --- \| --- \| \| Number of comparisons per family \| 3 \|  \|  \|  \|  \|  \|  \|  \| \| Alpha \| 0.05 \|  \|  \|  \|  \|  \|  \|  \| \|  \|  \|  \|  \|  \|  \|  \|  \|  \| \| Tukey's multiple comparisons test \| Mean Diff. \| 95.00% CI of diff. \| Significant? \| Summary \| Adjusted P Value \|  \|  \|  \| \|  \|  \|  \|  \|  \|  \|  \|  \|  \| \| Cont vs. Syn^A35T^/NOS^+/+^ \| 731755 \| -8383546 to 9847057 \| No \| ns \| 0.9794 \| A-B \|  \|  \| \| Cont vs. SynA^35T^/NOS-^/-^ \| 10199425 \| 570671 to 19828179 \| Yes \| * \| 0.0357 \| A-C \|  \|  \| \| Syn^A35T^/NOS^+/+^ vs. SynA^35T^/NOS-^/-^ \| 9467670 \| -504784 to 19440123 \| No \| ns \| 0.0660 \| B-C \|  \|  \| \|  \|  \|  \|  \|  \|  \|  \|  \|  \| \|  \|  \|  \|  \|  \|  \|  \|  \|  \| \| Test details \| Mean 1 \| Mean 2 \| Mean Diff. \| SE of diff. \| n1 \| n2 \| q \| DF \| \|  \|  \|  \|  \|  \|  \|  \|  \|  \| \| Cont vs. Syn^A35T^/NOS^+/+^ \| 11351192 \| 10619437 \| 731755 \| 3769009 \| 20 \| 17 \| 0.2746 \| 48 \| \| Cont vs. SynA^35T^/NOS-^/-^ \| 11351192 \| 1151767 \| 10199425 \| 3981312 \| 20 \| 14 \| 3.623 \| 48 \| \| Syn^A35T^/NOS^+/+^ vs. SynA^35T^/NOS-^/-^ \| 10619437 \| 1151767 \| 9467670 \| 4123425 \| 17 \| 14 \| 3.247 \| 48 \| |
| **Figure S3C: GFAP cells-DpMe** |
| \| Number of families \| 1 \|  \|  \|  \|  \|  \|  \|  \| \| --- \| --- \| --- \| --- \| --- \| --- \| --- \| --- \| --- \| \| Number of comparisons per family \| 3 \|  \|  \|  \|  \|  \|  \|  \| \| Alpha \| 0.05 \|  \|  \|  \|  \|  \|  \|  \| \|  \|  \|  \|  \|  \|  \|  \|  \|  \| \| Tukey's multiple comparisons test \| Mean Diff. \| 95.00% CI of diff. \| Significant? \| Summary \| Adjusted P Value \|  \|  \|  \| \|  \|  \|  \|  \|  \|  \|  \|  \|  \| \| Cont vs. Syn^A35T^/NOS^+/+^ \| 0 \| -6297197 to 6297197 \| No \| ns \| >0.9999 \| D-E \|  \|  \| \| Cont vs. SynA^35T^/NOS-^/-^ \| 9994527 \| 3315341 to 16673713 \| Yes \| ** \| 0.0019 \| D-F \|  \|  \| \| Syn^A35T^/NOS^+/+^ vs. SynA^35T^/NOS-^/-^ \| 9994527 \| 3315341 to 16673713 \| Yes \| ** \| 0.0019 \| E-F \|  \|  \| \|  \|  \|  \|  \|  \|  \|  \|  \|  \| \|  \|  \|  \|  \|  \|  \|  \|  \|  \| \| Test details \| Mean 1 \| Mean 2 \| Mean Diff. \| SE of diff. \| n1 \| n2 \| q \| DF \| \|  \|  \|  \|  \|  \|  \|  \|  \|  \| \| Cont vs. Syn^A35T^/NOS^+/+^ \| 10121640 \| 10121640 \| 0 \| 2611575 \| 20 \| 20 \| 0 \| 53 \| \| Cont vs. SynA^35T^/NOS-^/-^ \| 10121640 \| 127114 \| 9994527 \| 2769993 \| 20 \| 16 \| 5.103 \| 53 \| \| Syn^A35T^/NOS^+/+^ vs. SynA^35T^/NOS-^/-^ \| 10121640 \| 127114 \| 9994527 \| 2769993 \| 20 \| 16 \| 5.103 \| 53 \| |
| **Figure S3C: GFAP cells-Gi** |
| \| Number of families \| 1 \|  \|  \|  \|  \|  \|  \|  \| \| --- \| --- \| --- \| --- \| --- \| --- \| --- \| --- \| --- \| \| Number of comparisons per family \| 3 \|  \|  \|  \|  \|  \|  \|  \| \| Alpha \| 0.05 \|  \|  \|  \|  \|  \|  \|  \| \|  \|  \|  \|  \|  \|  \|  \|  \|  \| \| Tukey's multiple comparisons test \| Mean Diff. \| 95.00% CI of diff. \| Significant? \| Summary \| Adjusted P Value \|  \|  \|  \| \|  \|  \|  \|  \|  \|  \|  \|  \|  \| \| Cont vs. Syn^A35T^/NOS^+/+^ \| -5474313 \| -12774874 to 1826248 \| No \| ns \| 0.1762 \| G-H \|  \|  \| \| Cont vs. SynA^35T^/NOS-^/-^ \| 11887454 \| 3457492 to 20317416 \| Yes \| ** \| 0.0037 \| G-I \|  \|  \| \| Syn^A35T^/NOS^+/+^ vs. SynA^35T^/NOS-^/-^ \| 17361767 \| 8931805 to 25791729 \| Yes \| **** \| <0.0001 \| H-I \|  \|  \| \|  \|  \|  \|  \|  \|  \|  \|  \|  \| \|  \|  \|  \|  \|  \|  \|  \|  \|  \| \| Test details \| Mean 1 \| Mean 2 \| Mean Diff. \| SE of diff. \| n1 \| n2 \| q \| DF \| \|  \|  \|  \|  \|  \|  \|  \|  \|  \| \| Cont vs. Syn^A35T^/NOS^+/+^ \| 11969355 \| 17443668 \| -5474313 \| 3020603 \| 20 \| 20 \| 2.563 \| 49 \| \| Cont vs. SynA^35T^/NOS-^/-^ \| 11969355 \| 81901 \| 11887454 \| 3487892 \| 20 \| 12 \| 4.82 \| 49 \| \| Syn^A35T^/NOS^+/+^ vs. SynA^35T^/NOS-^/-^ \| 17443668 \| 81901 \| 17361767 \| 3487892 \| 20 \| 12 \| 7.04 \| 49 \| |
| **Figure S3D: GFAP % area-SN** |
| \| Number of families \| 1 \|  \|  \|  \|  \|  \|  \|  \| \| --- \| --- \| --- \| --- \| --- \| --- \| --- \| --- \| --- \| \| Number of comparisons per family \| 3 \|  \|  \|  \|  \|  \|  \|  \| \| Alpha \| 0.05 \|  \|  \|  \|  \|  \|  \|  \| \|  \|  \|  \|  \|  \|  \|  \|  \|  \| \| Tukey's multiple comparisons test \| Mean Diff. \| 95.00% CI of diff. \| Significant? \| Summary \| Adjusted P Value \|  \|  \|  \| \|  \|  \|  \|  \|  \|  \|  \|  \|  \| \| Cont vs. Syn^A35T^/NOS^+/+^ \| 0.3983 \| -1.156 to 1.953 \| No \| ns \| 0.8094 \| A-B \|  \|  \| \| Cont vs. SynA^35T^/NOS-^/-^ \| 1.479 \| -0.1696 to 3.128 \| No \| ns \| 0.0867 \| A-C \|  \|  \| \| Syn^A35T^/NOS^+/+^ vs. SynA^35T^/NOS-^/-^ \| 1.081 \| -0.6295 to 2.791 \| No \| ns \| 0.2860 \| B-C \|  \|  \| \|  \|  \|  \|  \|  \|  \|  \|  \|  \| \|  \|  \|  \|  \|  \|  \|  \|  \|  \| \| Test details \| Mean 1 \| Mean 2 \| Mean Diff. \| SE of diff. \| n1 \| n2 \| q \| DF \| \|  \|  \|  \|  \|  \|  \|  \|  \|  \| \| Cont vs. Syn^A35T^/NOS^+/+^ \| 1.621 \| 1.223 \| 0.3983 \| 0.6413 \| 19 \| 16 \| 0.8783 \| 45 \| \| Cont vs. SynA^35T^/NOS-^/-^ \| 1.621 \| 0.142 \| 1.479 \| 0.6803 \| 19 \| 13 \| 3.075 \| 45 \| \| Syn^A35T^/NOS^+/+^ vs. SynA^35T^/NOS-^/-^ \| 1.223 \| 0.142 \| 1.081 \| 0.7057 \| 16 \| 13 \| 2.166 \| 45 \| |
| **Figure S3D: GFAP % area -DpMe** |
| \| Number of families \| 1 \|  \|  \|  \|  \|  \|  \|  \| \| --- \| --- \| --- \| --- \| --- \| --- \| --- \| --- \| --- \| \| Number of comparisons per family \| 3 \|  \|  \|  \|  \|  \|  \|  \| \| Alpha \| 0.05 \|  \|  \|  \|  \|  \|  \|  \| \|  \|  \|  \|  \|  \|  \|  \|  \|  \| \| Tukey's multiple comparisons test \| Mean Diff. \| 95.00% CI of diff. \| Significant? \| Summary \| Adjusted P Value \|  \|  \|  \| \|  \|  \|  \|  \|  \|  \|  \|  \|  \| \| Cont vs. Syn^A35T^/NOS^+/+^ \| -14.6 \| -25.03 to -4.169 \| Yes \| ** \| 0.0040 \| D-E \|  \|  \| \| Cont vs. SynA^35T^/NOS-^/-^ \| 3.084 \| -7.523 to 13.69 \| No \| ns \| 0.7633 \| D-F \|  \|  \| \| Syn^A35T^/NOS^+/+^ vs. SynA^35T^/NOS-^/-^ \| 17.68 \| 6.67 to 28.7 \| Yes \| *** \| 0.0009 \| E-F \|  \|  \| \|  \|  \|  \|  \|  \|  \|  \|  \|  \| \|  \|  \|  \|  \|  \|  \|  \|  \|  \| \| Test details \| Mean 1 \| Mean 2 \| Mean Diff. \| SE of diff. \| n1 \| n2 \| q \| DF \| \|  \|  \|  \|  \|  \|  \|  \|  \|  \| \| Cont vs. Syn^A35T^/NOS^+/+^ \| 3.102 \| 17.7 \| -14.6 \| 4.319 \| 20 \| 17 \| 4.781 \| 50 \| \| Cont vs. SynA^35T^/NOS-^/-^ \| 3.102 \| 0.01827 \| 3.084 \| 4.391 \| 20 \| 16 \| 0.9931 \| 50 \| \| Syn^A35T^/NOS^+/+^ vs. SynA^35T^/NOS-^/-^ \| 17.7 \| 0.01827 \| 17.68 \| 4.56 \| 17 \| 16 \| 5.484 \| 50 \| |
| **Figure S3D: GFAP % area -Gi** |
| \| Number of families \| 1 \|  \|  \|  \|  \|  \|  \|  \| \| --- \| --- \| --- \| --- \| --- \| --- \| --- \| --- \| --- \| \| Number of comparisons per family \| 3 \|  \|  \|  \|  \|  \|  \|  \| \| Alpha \| 0.05 \|  \|  \|  \|  \|  \|  \|  \| \|  \|  \|  \|  \|  \|  \|  \|  \|  \| \| Tukey's multiple comparisons test \| Mean Diff. \| 95.00% CI of diff. \| Significant? \| Summary \| Adjusted P Value \|  \|  \|  \| \|  \|  \|  \|  \|  \|  \|  \|  \|  \| \| Cont vs. Syn^A35T^/NOS^+/+^ \| -35.94 \| -47.17 to -24.71 \| Yes \| **** \| <0.0001 \| G-H \|  \|  \| \| Cont vs. SynA^35T^/NOS-^/-^ \| 4.317 \| -8.645 to 17.28 \| No \| ns \| 0.7018 \| G-I \|  \|  \| \| Syn^A35T^/NOS^+/+^ vs. SynA^35T^/NOS-^/-^ \| 40.26 \| 27.29 to 53.22 \| Yes \| **** \| <0.0001 \| H-I \|  \|  \| \|  \|  \|  \|  \|  \|  \|  \|  \|  \| \|  \|  \|  \|  \|  \|  \|  \|  \|  \| \| Test details \| Mean 1 \| Mean 2 \| Mean Diff. \| SE of diff. \| n1 \| n2 \| q \| DF \| \|  \|  \|  \|  \|  \|  \|  \|  \|  \| \| Cont vs. Syn^A35T^/NOS^+/+^ \| 4.335 \| 40.27 \| -35.94 \| 4.645 \| 20 \| 20 \| 10.94 \| 49 \| \| Cont vs. SynA^35T^/NOS-^/-^ \| 4.335 \| 0.0177 \| 4.317 \| 5.363 \| 20 \| 12 \| 1.138 \| 49 \| \| Syn^A35T^/NOS^+/+^ vs. SynA^35T^/NOS-^/-^ \| 40.27 \| 0.0177 \| 40.26 \| 5.363 \| 20 \| 12 \| 10.61 \| 49 \| |
| **Figure S4B: Iba-1 fluorescence intensity-Cortex** |
| \| Number of families \| 1 \|  \|  \|  \|  \|  \|  \|  \| \| --- \| --- \| --- \| --- \| --- \| --- \| --- \| --- \| --- \| \| Number of comparisons per family \| 3 \|  \|  \|  \|  \|  \|  \|  \| \| Alpha \| 0.05 \|  \|  \|  \|  \|  \|  \|  \| \|  \|  \|  \|  \|  \|  \|  \|  \|  \| \| Tukey's multiple comparisons test \| Mean Diff. \| 95.00% CI of diff. \| Significant? \| Summary \| Adjusted P Value \|  \|  \|  \| \|  \|  \|  \|  \|  \|  \|  \|  \|  \| \| Cont vs. Syn^A35T^/NOS^+/+^ \| -3.692 \| -34.41 to 27.02 \| No \| ns \| 0.9546 \| A-B \|  \|  \| \| Cont vs. SynA^35T^/NOS-^/-^ \| 68.2 \| 34.79 to 101.6 \| Yes \| **** \| <0.0001 \| A-C \|  \|  \| \| Syn^A35T^/NOS^+/+^ vs. SynA^35T^/NOS-^/-^ \| 71.89 \| 38.12 to 105.7 \| Yes \| **** \| <0.0001 \| B-C \|  \|  \| \|  \|  \|  \|  \|  \|  \|  \|  \|  \| \|  \|  \|  \|  \|  \|  \|  \|  \|  \| \| Test details \| Mean 1 \| Mean 2 \| Mean Diff. \| SE of diff. \| n1 \| n2 \| q \| DF \| \|  \|  \|  \|  \|  \|  \|  \|  \|  \| \| Cont vs. Syn^A35T^/NOS^+/+^ \| 100 \| 103.7 \| -3.692 \| 12.72 \| 20 \| 19 \| 0.4106 \| 50 \| \| Cont vs. SynA^35T^/NOS-^/-^ \| 100 \| 31.8 \| 68.2 \| 13.83 \| 20 \| 14 \| 6.972 \| 50 \| \| Syn^A35T^/NOS^+/+^ vs. SynA^35T^/NOS-^/-^ \| 103.7 \| 31.8 \| 71.89 \| 13.98 \| 19 \| 14 \| 7.271 \| 50 \| |
| **Figure S4B: Iba-1 fluorescence intensity-CPu** |
| \| Number of families \| 1 \|  \|  \|  \|  \|  \|  \|  \| \| --- \| --- \| --- \| --- \| --- \| --- \| --- \| --- \| --- \| \| Number of comparisons per family \| 3 \|  \|  \|  \|  \|  \|  \|  \| \| Alpha \| 0.05 \|  \|  \|  \|  \|  \|  \|  \| \|  \|  \|  \|  \|  \|  \|  \|  \|  \| \| Tukey's multiple comparisons test \| Mean Diff. \| 95.00% CI of diff. \| Significant? \| Summary \| Adjusted P Value \|  \|  \|  \| \|  \|  \|  \|  \|  \|  \|  \|  \|  \| \| Cont vs. Syn^A35T^/NOS^+/+^ \| -7.789 \| -36.95 to 21.38 \| No \| ns \| 0.7960 \| D-E \|  \|  \| \| Cont vs. SynA^35T^/NOS-^/-^ \| 63.55 \| 31.83 to 95.27 \| Yes \| **** \| <0.0001 \| D-F \|  \|  \| \| Syn^A35T^/NOS^+/+^ vs. SynA^35T^/NOS-^/-^ \| 71.34 \| 39.27 to 103.4 \| Yes \| **** \| <0.0001 \| E-F \|  \|  \| \|  \|  \|  \|  \|  \|  \|  \|  \|  \| \|  \|  \|  \|  \|  \|  \|  \|  \|  \| \| Test details \| Mean 1 \| Mean 2 \| Mean Diff. \| SE of diff. \| n1 \| n2 \| q \| DF \| \|  \|  \|  \|  \|  \|  \|  \|  \|  \| \| Cont vs. Syn^A35T^/NOS^+/+^ \| 100 \| 107.8 \| -7.789 \| 12.07 \| 20 \| 19 \| 0.9123 \| 50 \| \| Cont vs. SynA^35T^/NOS-^/-^ \| 100 \| 36.45 \| 63.55 \| 13.13 \| 20 \| 14 \| 6.843 \| 50 \| \| Syn^A35T^/NOS^+/+^ vs. SynA^35T^/NOS-^/-^ \| 107.8 \| 36.45 \| 71.34 \| 13.27 \| 19 \| 14 \| 7.6 \| 50 \| |
| **Figure S4B: Iba-1 fluorescence intensity-Hippo** |
| \| Number of families \| 1 \|  \|  \|  \|  \|  \|  \|  \| \| --- \| --- \| --- \| --- \| --- \| --- \| --- \| --- \| --- \| \| Number of comparisons per family \| 3 \|  \|  \|  \|  \|  \|  \|  \| \| Alpha \| 0.05 \|  \|  \|  \|  \|  \|  \|  \| \|  \|  \|  \|  \|  \|  \|  \|  \|  \| \| Tukey's multiple comparisons test \| Mean Diff. \| 95.00% CI of diff. \| Significant? \| Summary \| Adjusted P Value \|  \|  \|  \| \|  \|  \|  \|  \|  \|  \|  \|  \|  \| \| Cont vs. Syn^A35T^/NOS^+/+^ \| 13.03 \| -20.89 to 46.95 \| No \| ns \| 0.6249 \| G-H \|  \|  \| \| Cont vs. SynA^35T^/NOS-^/-^ \| 57.81 \| 20.61 to 95 \| Yes \| ** \| 0.0013 \| G-I \|  \|  \| \| Syn^A35T^/NOS^+/+^ vs. SynA^35T^/NOS-^/-^ \| 44.78 \| 6.779 to 82.78 \| Yes \| * \| 0.0173 \| H-I \|  \|  \| \|  \|  \|  \|  \|  \|  \|  \|  \|  \| \|  \|  \|  \|  \|  \|  \|  \|  \|  \| \| Test details \| Mean 1 \| Mean 2 \| Mean Diff. \| SE of diff. \| n1 \| n2 \| q \| DF \| \|  \|  \|  \|  \|  \|  \|  \|  \|  \| \| Cont vs. Syn^A35T^/NOS^+/+^ \| 100 \| 86.97 \| 13.03 \| 14.02 \| 20 \| 18 \| 1.314 \| 48 \| \| Cont vs. SynA^35T^/NOS-^/-^ \| 100 \| 42.19 \| 57.81 \| 15.38 \| 20 \| 13 \| 5.316 \| 48 \| \| Syn^A35T^/NOS^+/+^ vs. SynA^35T^/NOS-^/-^ \| 86.97 \| 42.19 \| 44.78 \| 15.71 \| 18 \| 13 \| 4.03 \| 48 \| |
| **Figure S4B: Iba-1 cells-Cortex** |
| \| Number of families \| 1 \|  \|  \|  \|  \|  \|  \|  \| \| --- \| --- \| --- \| --- \| --- \| --- \| --- \| --- \| --- \| \| Number of comparisons per family \| 3 \|  \|  \|  \|  \|  \|  \|  \| \| Alpha \| 0.05 \|  \|  \|  \|  \|  \|  \|  \| \|  \|  \|  \|  \|  \|  \|  \|  \|  \| \| Tukey's multiple comparisons test \| Mean Diff. \| 95.00% CI of diff. \| Significant? \| Summary \| Adjusted P Value \|  \|  \|  \| \|  \|  \|  \|  \|  \|  \|  \|  \|  \| \| Cont vs. Syn^A35T^/NOS^+/+^ \| 1357619 \| -3457133 to 6172372 \| No \| ns \| 0.7755 \| A-B \|  \|  \| \| Cont vs. SynA^35T^/NOS-^/-^ \| 9636796 \| 4399659 to 14873932 \| Yes \| *** \| 0.0001 \| A-C \|  \|  \| \| Syn^A35T^/NOS^+/+^ vs. SynA^35T^/NOS-^/-^ \| 8279176 \| 2985595 to 13572758 \| Yes \| ** \| 0.0012 \| B-C \|  \|  \| \|  \|  \|  \|  \|  \|  \|  \|  \|  \| \|  \|  \|  \|  \|  \|  \|  \|  \|  \| \| Test details \| Mean 1 \| Mean 2 \| Mean Diff. \| SE of diff. \| n1 \| n2 \| q \| DF \| \|  \|  \|  \|  \|  \|  \|  \|  \|  \| \| Cont vs. Syn^A35T^/NOS^+/+^ \| 13603976 \| 12246357 \| 1357619 \| 1993339 \| 20 \| 19 \| 0.9632 \| 50 \| \| Cont vs. SynA^35T^/NOS-^/-^ \| 13603976 \| 3967181 \| 9636796 \| 2168209 \| 20 \| 14 \| 6.286 \| 50 \| \| Syn^A35T^/NOS^+/+^ vs. SynA^35T^/NOS-^/-^ \| 12246357 \| 3967181 \| 8279176 \| 2191577 \| 19 \| 14 \| 5.343 \| 50 \| |
| **Figure S4B: Iba-1 cells-CPu** |
| \| Number of families \| 1 \|  \|  \|  \|  \|  \|  \|  \| \| --- \| --- \| --- \| --- \| --- \| --- \| --- \| --- \| --- \| \| Number of comparisons per family \| 3 \|  \|  \|  \|  \|  \|  \|  \| \| Alpha \| 0.05 \|  \|  \|  \|  \|  \|  \|  \| \|  \|  \|  \|  \|  \|  \|  \|  \|  \| \| Tukey's multiple comparisons test \| Mean Diff. \| 95.00% CI of diff. \| Significant? \| Summary \| Adjusted P Value \|  \|  \|  \| \|  \|  \|  \|  \|  \|  \|  \|  \|  \| \| Cont vs. Syn^A35T^/NOS^+/+^ \| 492833 \| -2012720 to 2998387 \| No \| ns \| 0.8833 \| D-E \|  \|  \| \| Cont vs. SynA^35T^/NOS-^/-^ \| 5815228 \| 3089870 to 8540586 \| Yes \| **** \| <0.0001 \| D-F \|  \|  \| \| Syn^A35T^/NOS^+/+^ vs. SynA^35T^/NOS-^/-^ \| 5322395 \| 2567663 to 8077126 \| Yes \| **** \| <0.0001 \| E-F \|  \|  \| \|  \|  \|  \|  \|  \|  \|  \|  \|  \| \|  \|  \|  \|  \|  \|  \|  \|  \|  \| \| Test details \| Mean 1 \| Mean 2 \| Mean Diff. \| SE of diff. \| n1 \| n2 \| q \| DF \| \|  \|  \|  \|  \|  \|  \|  \|  \|  \| \| Cont vs. Syn^A35T^/NOS^+/+^ \| 9644219 \| 9151385 \| 492833 \| 1037315 \| 20 \| 19 \| 0.6719 \| 50 \| \| Cont vs. SynA^35T^/NOS-^/-^ \| 9644219 \| 3828991 \| 5815228 \| 1128316 \| 20 \| 14 \| 7.289 \| 50 \| \| Syn^A35T^/NOS^+/+^ vs. SynA^35T^/NOS-^/-^ \| 9151385 \| 3828991 \| 5322395 \| 1140477 \| 19 \| 14 \| 6.6 \| 50 \| |
| **Figure S4B: Iba-1 cells-Hippo** |
| \| Number of families \| 1 \|  \|  \|  \|  \|  \|  \|  \| \| --- \| --- \| --- \| --- \| --- \| --- \| --- \| --- \| --- \| \| Number of comparisons per family \| 3 \|  \|  \|  \|  \|  \|  \|  \| \| Alpha \| 0.05 \|  \|  \|  \|  \|  \|  \|  \| \|  \|  \|  \|  \|  \|  \|  \|  \|  \| \| Tukey's multiple comparisons test \| Mean Diff. \| 95.00% CI of diff. \| Significant? \| Summary \| Adjusted P Value \|  \|  \|  \| \|  \|  \|  \|  \|  \|  \|  \|  \|  \| \| Cont vs. Syn^A35T^/NOS^+/+^ \| -2603395 \| -6698969 to 1492179 \| No \| ns \| 0.2828 \| G-H \|  \|  \| \| Cont vs. SynA^35T^/NOS-^/-^ \| 2983353 \| -1507662 to 7474368 \| No \| ns \| 0.2527 \| G-I \|  \|  \| \| Syn^A35T^/NOS^+/+^ vs. SynA^35T^/NOS-^/-^ \| 5586748 \| 998497 to 10174999 \| Yes \| * \| 0.0135 \| H-I \|  \|  \| \|  \|  \|  \|  \|  \|  \|  \|  \|  \| \|  \|  \|  \|  \|  \|  \|  \|  \|  \| \| Test details \| Mean 1 \| Mean 2 \| Mean Diff. \| SE of diff. \| n1 \| n2 \| q \| DF \| \|  \|  \|  \|  \|  \|  \|  \|  \|  \| \| Cont vs. Syn^A35T^/NOS^+/+^ \| 6354981 \| 8958376 \| -2603395 \| 1693444 \| 20 \| 18 \| 2.174 \| 48 \| \| Cont vs. SynA^35T^/NOS-^/-^ \| 6354981 \| 3371628 \| 2983353 \| 1856952 \| 20 \| 13 \| 2.272 \| 48 \| \| Syn^A35T^/NOS^+/+^ vs. SynA^35T^/NOS-^/-^ \| 8958376 \| 3371628 \| 5586748 \| 1897157 \| 18 \| 13 \| 4.165 \| 48 \| |
| **Figure S4B: Iba-1 % area-Cortex** |
| \| Number of families \| 1 \|  \|  \|  \|  \|  \|  \|  \| \| --- \| --- \| --- \| --- \| --- \| --- \| --- \| --- \| --- \| \| Number of comparisons per family \| 3 \|  \|  \|  \|  \|  \|  \|  \| \| Alpha \| 0.05 \|  \|  \|  \|  \|  \|  \|  \| \|  \|  \|  \|  \|  \|  \|  \|  \|  \| \| Tukey's multiple comparisons test \| Mean Diff. \| 95.00% CI of diff. \| Significant? \| Summary \| Adjusted P Value \|  \|  \|  \| \|  \|  \|  \|  \|  \|  \|  \|  \|  \| \| Cont vs. Syn^A35T^/NOS^+/+^ \| -0.3273 \| -1.437 to 0.7829 \| No \| ns \| 0.7575 \| A-B \|  \|  \| \| Cont vs. SynA^35T^/NOS-^/-^ \| 1.915 \| 0.707 to 3.122 \| Yes \| ** \| 0.0010 \| A-C \|  \|  \| \| Syn^A35T^/NOS^+/+^ vs. SynA^35T^/NOS-^/-^ \| 2.242 \| 1.021 to 3.462 \| Yes \| *** \| 0.0001 \| B-C \|  \|  \| \|  \|  \|  \|  \|  \|  \|  \|  \|  \| \|  \|  \|  \|  \|  \|  \|  \|  \|  \| \| Test details \| Mean 1 \| Mean 2 \| Mean Diff. \| SE of diff. \| n1 \| n2 \| q \| DF \| \|  \|  \|  \|  \|  \|  \|  \|  \|  \| \| Cont vs. Syn^A35T^/NOS^+/+^ \| 2.859 \| 3.187 \| -0.3273 \| 0.4596 \| 20 \| 19 \| 1.007 \| 50 \| \| Cont vs. SynA^35T^/NOS-^/-^ \| 2.859 \| 0.9449 \| 1.915 \| 0.4999 \| 20 \| 14 \| 5.416 \| 50 \| \| Syn^A35T^/NOS^+/+^ vs. SynA^35T^/NOS-^/-^ \| 3.187 \| 0.9449 \| 2.242 \| 0.5053 \| 19 \| 14 \| 6.274 \| 50 \| |
| **Figure S4B: Iba-1 % area-CPu** |
| \| Number of families \| 1 \|  \|  \|  \|  \|  \|  \|  \| \| --- \| --- \| --- \| --- \| --- \| --- \| --- \| --- \| --- \| \| Number of comparisons per family \| 3 \|  \|  \|  \|  \|  \|  \|  \| \| Alpha \| 0.05 \|  \|  \|  \|  \|  \|  \|  \| \|  \|  \|  \|  \|  \|  \|  \|  \|  \| \| Tukey's multiple comparisons test \| Mean Diff. \| 95.00% CI of diff. \| Significant? \| Summary \| Adjusted P Value \|  \|  \|  \| \|  \|  \|  \|  \|  \|  \|  \|  \|  \| \| Cont vs. Syn^A35T^/NOS^+/+^ \| 0.01517 \| -0.5643 to 0.5946 \| No \| ns \| 0.9978 \| D-E \|  \|  \| \| Cont vs. SynA^35T^/NOS-^/-^ \| 1.291 \| 0.6606 to 1.921 \| Yes \| **** \| <0.0001 \| D-F \|  \|  \| \| Syn^A35T^/NOS^+/+^ vs. SynA^35T^/NOS-^/-^ \| 1.276 \| 0.6386 to 1.913 \| Yes \| **** \| <0.0001 \| E-F \|  \|  \| \|  \|  \|  \|  \|  \|  \|  \|  \|  \| \|  \|  \|  \|  \|  \|  \|  \|  \|  \| \| Test details \| Mean 1 \| Mean 2 \| Mean Diff. \| SE of diff. \| n1 \| n2 \| q \| DF \| \|  \|  \|  \|  \|  \|  \|  \|  \|  \| \| Cont vs. Syn^A35T^/NOS^+/+^ \| 2.052 \| 2.037 \| 0.01517 \| 0.2399 \| 20 \| 19 \| 0.08944 \| 50 \| \| Cont vs. SynA^35T^/NOS-^/-^ \| 2.052 \| 0.7609 \| 1.291 \| 0.2609 \| 20 \| 14 \| 6.996 \| 50 \| \| Syn^A35T^/NOS^+/+^ vs. SynA^35T^/NOS-^/-^ \| 2.037 \| 0.7609 \| 1.276 \| 0.2637 \| 19 \| 14 \| 6.84 \| 50 \| |
| **Figure S4B: Iba-1 % area-Hippo** |
| \| Number of families \| 1 \|  \|  \|  \|  \|  \|  \|  \| \| --- \| --- \| --- \| --- \| --- \| --- \| --- \| --- \| --- \| \| Number of comparisons per family \| 3 \|  \|  \|  \|  \|  \|  \|  \| \| Alpha \| 0.05 \|  \|  \|  \|  \|  \|  \|  \| \|  \|  \|  \|  \|  \|  \|  \|  \|  \| \| Tukey's multiple comparisons test \| Mean Diff. \| 95.00% CI of diff. \| Significant? \| Summary \| Adjusted P Value \|  \|  \|  \| \|  \|  \|  \|  \|  \|  \|  \|  \|  \| \| Cont vs. Syn^A35T^/NOS^+/+^ \| -0.6489 \| -1.636 to 0.3378 \| No \| ns \| 0.2595 \| G-H \|  \|  \| \| Cont vs. SynA^35T^/NOS-^/-^ \| 0.7688 \| -0.3131 to 1.851 \| No \| ns \| 0.2088 \| G-I \|  \|  \| \| Syn^A35T^/NOS^+/+^ vs. SynA^35T^/NOS-^/-^ \| 1.418 \| 0.3124 to 2.523 \| Yes \| ** \| 0.0089 \| H-I \|  \|  \| \|  \|  \|  \|  \|  \|  \|  \|  \|  \| \|  \|  \|  \|  \|  \|  \|  \|  \|  \| \| Test details \| Mean 1 \| Mean 2 \| Mean Diff. \| SE of diff. \| n1 \| n2 \| q \| DF \| \|  \|  \|  \|  \|  \|  \|  \|  \|  \| \| Cont vs. Syn^A35T^/NOS^+/+^ \| 1.391 \| 2.04 \| -0.6489 \| 0.408 \| 20 \| 18 \| 2.249 \| 48 \| \| Cont vs. SynA^35T^/NOS-^/-^ \| 1.391 \| 0.6225 \| 0.7688 \| 0.4474 \| 20 \| 13 \| 2.43 \| 48 \| \| Syn^A35T^/NOS^+/+^ vs. SynA^35T^/NOS-^/-^ \| 2.04 \| 0.6225 \| 1.418 \| 0.4571 \| 18 \| 13 \| 4.387 \| 48 \| |
| **Figure S5B: GFAP fluorescence intensity-Cortex** |
| \| Number of families \| 1 \|  \|  \|  \|  \|  \|  \|  \| \| --- \| --- \| --- \| --- \| --- \| --- \| --- \| --- \| --- \| \| Number of comparisons per family \| 3 \|  \|  \|  \|  \|  \|  \|  \| \| Alpha \| 0.05 \|  \|  \|  \|  \|  \|  \|  \| \|  \|  \|  \|  \|  \|  \|  \|  \|  \| \| Tukey's multiple comparisons test \| Mean Diff. \| 95.00% CI of diff. \| Significant? \| Summary \| Adjusted P Value \|  \|  \|  \| \|  \|  \|  \|  \|  \|  \|  \|  \|  \| \| Cont vs. Syn^A35T^/NOS^+/+^ \| -0.5063 \| -31.95 to 30.94 \| No \| ns \| 0.9992 \| A-B \|  \|  \| \| Cont vs. SynA^35T^/NOS-^/-^ \| 54.7 \| 20.5 to 88.9 \| Yes \| *** \| 0.0009 \| A-C \|  \|  \| \| Syn^A35T^/NOS^+/+^ vs. SynA^35T^/NOS-^/-^ \| 55.21 \| 20.64 to 89.77 \| Yes \| *** \| 0.0009 \| B-C \|  \|  \| \|  \|  \|  \|  \|  \|  \|  \|  \|  \| \|  \|  \|  \|  \|  \|  \|  \|  \|  \| \| Test details \| Mean 1 \| Mean 2 \| Mean Diff. \| SE of diff. \| n1 \| n2 \| q \| DF \| \|  \|  \|  \|  \|  \|  \|  \|  \|  \| \| Cont vs. Syn^A35T^/NOS^+/+^ \| 100 \| 100.5 \| -0.5063 \| 13.02 \| 20 \| 19 \| 0.05501 \| 50 \| \| Cont vs. SynA^35T^/NOS-^/-^ \| 100 \| 45.3 \| 54.7 \| 14.16 \| 20 \| 14 \| 5.463 \| 50 \| \| Syn^A35T^/NOS^+/+^ vs. SynA^35T^/NOS-^/-^ \| 100.5 \| 45.3 \| 55.21 \| 14.31 \| 19 \| 14 \| 5.455 \| 50 \| |
| **Figure S5B: GFAP fluorescence intensity-CPu** |
| \| Number of families \| 1 \|  \|  \|  \|  \|  \|  \|  \| \| --- \| --- \| --- \| --- \| --- \| --- \| --- \| --- \| --- \| \| Number of comparisons per family \| 3 \|  \|  \|  \|  \|  \|  \|  \| \| Alpha \| 0.05 \|  \|  \|  \|  \|  \|  \|  \| \|  \|  \|  \|  \|  \|  \|  \|  \|  \| \| Tukey's multiple comparisons test \| Mean Diff. \| 95.00% CI of diff. \| Significant? \| Summary \| Adjusted P Value \|  \|  \|  \| \|  \|  \|  \|  \|  \|  \|  \|  \|  \| \| Cont vs. Syn^A35T^/NOS^+/+^ \| -2.862 \| -35.57 to 29.85 \| No \| ns \| 0.9757 \| D-E \|  \|  \| \| Cont vs. SynA^35T^/NOS-^/-^ \| 59.59 \| 24.01 to 95.16 \| Yes \| *** \| 0.0005 \| D-F \|  \|  \| \| Syn^A35T^/NOS^+/+^ vs. SynA^35T^/NOS-^/-^ \| 62.45 \| 26.49 to 98.41 \| Yes \| *** \| 0.0003 \| E-F \|  \|  \| \|  \|  \|  \|  \|  \|  \|  \|  \|  \| \|  \|  \|  \|  \|  \|  \|  \|  \|  \| \| Test details \| Mean 1 \| Mean 2 \| Mean Diff. \| SE of diff. \| n1 \| n2 \| q \| DF \| \|  \|  \|  \|  \|  \|  \|  \|  \|  \| \| Cont vs. Syn^A35T^/NOS^+/+^ \| 100 \| 102.9 \| -2.862 \| 13.54 \| 20 \| 19 \| 0.2989 \| 50 \| \| Cont vs. SynA^35T^/NOS-^/-^ \| 100 \| 40.41 \| 59.59 \| 14.73 \| 20 \| 14 \| 5.721 \| 50 \| \| Syn^A35T^/NOS^+/+^ vs. SynA^35T^/NOS-^/-^ \| 102.9 \| 40.41 \| 62.45 \| 14.89 \| 19 \| 14 \| 5.932 \| 50 \| |
| **Figure S5B: GFAP fluorescence intensity-Hippo** |
| \| Number of families \| 1 \|  \|  \|  \|  \|  \|  \|  \| \| --- \| --- \| --- \| --- \| --- \| --- \| --- \| --- \| --- \| \| Number of comparisons per family \| 3 \|  \|  \|  \|  \|  \|  \|  \| \| Alpha \| 0.05 \|  \|  \|  \|  \|  \|  \|  \| \|  \|  \|  \|  \|  \|  \|  \|  \|  \| \| Tukey's multiple comparisons test \| Mean Diff. \| 95.00% CI of diff. \| Significant? \| Summary \| Adjusted P Value \|  \|  \|  \| \|  \|  \|  \|  \|  \|  \|  \|  \|  \| \| Cont vs. Syn^A35T^/NOS^+/+^ \| -3.044 \| -31.6 to 25.51 \| No \| ns \| 0.9640 \| G-H \|  \|  \| \| Cont vs. SynA^35T^/NOS-^/-^ \| 62.14 \| 30.9 to 93.39 \| Yes \| **** \| <0.0001 \| G-I \|  \|  \| \| Syn^A35T^/NOS^+/+^ vs. SynA^35T^/NOS-^/-^ \| 65.19 \| 33.59 to 96.79 \| Yes \| **** \| <0.0001 \| H-I \|  \|  \| \|  \|  \|  \|  \|  \|  \|  \|  \|  \| \|  \|  \|  \|  \|  \|  \|  \|  \|  \| \| Test details \| Mean 1 \| Mean 2 \| Mean Diff. \| SE of diff. \| n1 \| n2 \| q \| DF \| \|  \|  \|  \|  \|  \|  \|  \|  \|  \| \| Cont vs. Syn^A35T^/NOS^+/+^ \| 100 \| 103 \| -3.044 \| 11.8 \| 19 \| 18 \| 0.3648 \| 47 \| \| Cont vs. SynA^35T^/NOS-^/-^ \| 100 \| 37.86 \| 62.14 \| 12.91 \| 19 \| 13 \| 6.807 \| 47 \| \| Syn^A35T^/NOS^+/+^ vs. SynA^35T^/NOS-^/-^ \| 103 \| 37.86 \| 65.19 \| 13.06 \| 18 \| 13 \| 7.061 \| 47 \| |
| **Figure S5B: GFAP cells-Cortex** |
| \| Number of families \| 1 \|  \|  \|  \|  \|  \|  \|  \| \| --- \| --- \| --- \| --- \| --- \| --- \| --- \| --- \| --- \| \| Number of comparisons per family \| 3 \|  \|  \|  \|  \|  \|  \|  \| \| Alpha \| 0.05 \|  \|  \|  \|  \|  \|  \|  \| \|  \|  \|  \|  \|  \|  \|  \|  \|  \| \| Tukey's multiple comparisons test \| Mean Diff. \| 95.00% CI of diff. \| Significant? \| Summary \| Adjusted P Value \|  \|  \|  \| \|  \|  \|  \|  \|  \|  \|  \|  \|  \| \| Cont vs. Syn^A35T^/NOS^+/+^ \| -4980671 \| -12189930 to 2228588 \| No \| ns \| 0.2272 \| A-B \|  \|  \| \| Cont vs. SynA^35T^/NOS-^/-^ \| 3758977 \| -4082730 to 11600683 \| No \| ns \| 0.4837 \| A-C \|  \|  \| \| Syn^A35T^/NOS^+/+^ vs. SynA^35T^/NOS-^/-^ \| 8739648 \| 813425 to 16665871 \| Yes \| * \| 0.0276 \| B-C \|  \|  \| \|  \|  \|  \|  \|  \|  \|  \|  \|  \| \|  \|  \|  \|  \|  \|  \|  \|  \|  \| \| Test details \| Mean 1 \| Mean 2 \| Mean Diff. \| SE of diff. \| n1 \| n2 \| q \| DF \| \|  \|  \|  \|  \|  \|  \|  \|  \|  \| \| Cont vs. Syn^A35T^/NOS^+/+^ \| 3842206 \| 8822877 \| -4980671 \| 2984681 \| 20 \| 19 \| 2.36 \| 50 \| \| Cont vs. SynA^35T^/NOS-^/-^ \| 3842206 \| 83230 \| 3758977 \| 3246518 \| 20 \| 14 \| 1.637 \| 50 \| \| Syn^A35T^/NOS^+/+^ vs. SynA^35T^/NOS-^/-^ \| 8822877 \| 83230 \| 8739648 \| 3281508 \| 19 \| 14 \| 3.766 \| 50 \| |
| **Figure S5B: GFAP cells-CPu** |
| \| Number of families \| 1 \|  \|  \|  \|  \|  \|  \|  \| \| --- \| --- \| --- \| --- \| --- \| --- \| --- \| --- \| --- \| \| Number of comparisons per family \| 3 \|  \|  \|  \|  \|  \|  \|  \| \| Alpha \| 0.05 \|  \|  \|  \|  \|  \|  \|  \| \|  \|  \|  \|  \|  \|  \|  \|  \|  \| \| Tukey's multiple comparisons test \| Mean Diff. \| 95.00% CI of diff. \| Significant? \| Summary \| Adjusted P Value \|  \|  \|  \| \|  \|  \|  \|  \|  \|  \|  \|  \|  \| \| Cont vs. Syn^A35T^/NOS^+/+^ \| -4679263 \| -8875825 to -482700 \| Yes \| * \| 0.0256 \| D-E \|  \|  \| \| Cont vs. SynA^35T^/NOS-^/-^ \| 1640088 \| -2924626 to 6204802 \| No \| ns \| 0.6629 \| D-F \|  \|  \| \| Syn^A35T^/NOS^+/+^ vs. SynA^35T^/NOS-^/-^ \| 6319351 \| 1705439 to 10933263 \| Yes \| ** \| 0.0049 \| E-F \|  \|  \| \|  \|  \|  \|  \|  \|  \|  \|  \|  \| \|  \|  \|  \|  \|  \|  \|  \|  \|  \| \| Test details \| Mean 1 \| Mean 2 \| Mean Diff. \| SE of diff. \| n1 \| n2 \| q \| DF \| \|  \|  \|  \|  \|  \|  \|  \|  \|  \| \| Cont vs. Syn^A35T^/NOS^+/+^ \| 1732647 \| 6411909 \| -4679263 \| 1737404 \| 20 \| 19 \| 3.809 \| 50 \| \| Cont vs. SynA^35T^/NOS-^/-^ \| 1732647 \| 92558 \| 1640088 \| 1889822 \| 20 \| 14 \| 1.227 \| 50 \| \| Syn^A35T^/NOS^+/+^ vs. SynA^35T^/NOS-^/-^ \| 6411909 \| 92558 \| 6319351 \| 1910190 \| 19 \| 14 \| 4.679 \| 50 \| |
| **Figure S5B: GFAP cells-Hippo** |
| \| Number of families \| 1 \|  \|  \|  \|  \|  \|  \|  \| \| --- \| --- \| --- \| --- \| --- \| --- \| --- \| --- \| --- \| \| Number of comparisons per family \| 3 \|  \|  \|  \|  \|  \|  \|  \| \| Alpha \| 0.05 \|  \|  \|  \|  \|  \|  \|  \| \|  \|  \|  \|  \|  \|  \|  \|  \|  \| \| Tukey's multiple comparisons test \| Mean Diff. \| 95.00% CI of diff. \| Significant? \| Summary \| Adjusted P Value \|  \|  \|  \| \|  \|  \|  \|  \|  \|  \|  \|  \|  \| \| Cont vs. Syn^A35T^/NOS^+/+^ \| -11841997 \| -22941560 to -742433 \| Yes \| * \| 0.0341 \| G-H \|  \|  \| \| Cont vs. SynA^35T^/NOS-^/-^ \| 6875065 \| -5271251 to 19021382 \| No \| ns \| 0.3647 \| G-I \|  \|  \| \| Syn^A35T^/NOS^+/+^ vs. SynA^35T^/NOS-^/-^ \| 18717062 \| 6434442 to 30999681 \| Yes \| ** \| 0.0017 \| H-I \|  \|  \| \|  \|  \|  \|  \|  \|  \|  \|  \|  \| \|  \|  \|  \|  \|  \|  \|  \|  \|  \| \| Test details \| Mean 1 \| Mean 2 \| Mean Diff. \| SE of diff. \| n1 \| n2 \| q \| DF \| \|  \|  \|  \|  \|  \|  \|  \|  \|  \| \| Cont vs. Syn^A35T^/NOS^+/+^ \| 10385375 \| 22227372 \| -11841997 \| 4586366 \| 19 \| 18 \| 3.651 \| 47 \| \| Cont vs. SynA^35T^/NOS-^/-^ \| 10385375 \| 3510310 \| 6875065 \| 5018887 \| 19 \| 13 \| 1.937 \| 47 \| \| Syn^A35T^/NOS^+/+^ vs. SynA^35T^/NOS-^/-^ \| 22227372 \| 3510310 \| 18717062 \| 5075208 \| 18 \| 13 \| 5.216 \| 47 \| |
| **Figure S5B: GFAP % area-Cortex** |
| \| Number of families \| 1 \|  \|  \|  \|  \|  \|  \|  \| \| --- \| --- \| --- \| --- \| --- \| --- \| --- \| --- \| --- \| \| Number of comparisons per family \| 3 \|  \|  \|  \|  \|  \|  \|  \| \| Alpha \| 0.05 \|  \|  \|  \|  \|  \|  \|  \| \|  \|  \|  \|  \|  \|  \|  \|  \|  \| \| Tukey's multiple comparisons test \| Mean Diff. \| 95.00% CI of diff. \| Significant? \| Summary \| Adjusted P Value \|  \|  \|  \| \|  \|  \|  \|  \|  \|  \|  \|  \|  \| \| Cont vs. Syn^A35T^/NOS^+/+^ \| -0.6267 \| -1.617 to 0.3638 \| No \| ns \| 0.2866 \| A-B \|  \|  \| \| Cont vs. SynA^35T^/NOS-^/-^ \| 0.3841 \| -0.6932 to 1.461 \| No \| ns \| 0.6670 \| A-C \|  \|  \| \| Syn^A35T^/NOS^+/+^ vs. SynA^35T^/NOS-^/-^ \| 1.011 \| -0.07818 to 2.1 \| No \| ns \| 0.0739 \| B-C \|  \|  \| \|  \|  \|  \|  \|  \|  \|  \|  \|  \| \|  \|  \|  \|  \|  \|  \|  \|  \|  \| \| Test details \| Mean 1 \| Mean 2 \| Mean Diff. \| SE of diff. \| n1 \| n2 \| q \| DF \| \|  \|  \|  \|  \|  \|  \|  \|  \|  \| \| Cont vs. Syn^A35T^/NOS^+/+^ \| 0.4066 \| 1.033 \| -0.6267 \| 0.4101 \| 20 \| 19 \| 2.161 \| 50 \| \| Cont vs. SynA^35T^/NOS-^/-^ \| 0.4066 \| 0.02243 \| 0.3841 \| 0.446 \| 20 \| 14 \| 1.218 \| 50 \| \| Syn^A35T^/NOS^+/+^ vs. SynA^35T^/NOS-^/-^ \| 1.033 \| 0.02243 \| 1.011 \| 0.4508 \| 19 \| 14 \| 3.171 \| 50 \| |
| **Figure S5B: GFAP % area-CPu** |
| \| Number of families \| 1 \|  \|  \|  \|  \|  \|  \|  \| \| --- \| --- \| --- \| --- \| --- \| --- \| --- \| --- \| --- \| \| Number of comparisons per family \| 3 \|  \|  \|  \|  \|  \|  \|  \| \| Alpha \| 0.05 \|  \|  \|  \|  \|  \|  \|  \| \|  \|  \|  \|  \|  \|  \|  \|  \|  \| \| Tukey's multiple comparisons test \| Mean Diff. \| 95.00% CI of diff. \| Significant? \| Summary \| Adjusted P Value \|  \|  \|  \| \|  \|  \|  \|  \|  \|  \|  \|  \|  \| \| Cont vs. Syn^A35T^/NOS^+/+^ \| -0.4586 \| -0.8523 to -0.0649 \| Yes \| * \| 0.0188 \| D-E \|  \|  \| \| Cont vs. SynA^35T^/NOS-^/-^ \| 0.1023 \| -0.326 to 0.5305 \| No \| ns \| 0.8330 \| D-F \|  \|  \| \| Syn^A35T^/NOS^+/+^ vs. SynA^35T^/NOS-^/-^ \| 0.5609 \| 0.128 to 0.9938 \| Yes \| ** \| 0.0081 \| E-F \|  \|  \| \|  \|  \|  \|  \|  \|  \|  \|  \|  \| \|  \|  \|  \|  \|  \|  \|  \|  \|  \| \| Test details \| Mean 1 \| Mean 2 \| Mean Diff. \| SE of diff. \| n1 \| n2 \| q \| DF \| \|  \|  \|  \|  \|  \|  \|  \|  \|  \| \| Cont vs. Syn^A35T^/NOS^+/+^ \| 0.117 \| 0.5756 \| -0.4586 \| 0.163 \| 20 \| 19 \| 3.979 \| 50 \| \| Cont vs. SynA^35T^/NOS-^/-^ \| 0.117 \| 0.01471 \| 0.1023 \| 0.1773 \| 20 \| 14 \| 0.8158 \| 50 \| \| Syn^A35T^/NOS^+/+^ vs. SynA^35T^/NOS-^/-^ \| 0.5756 \| 0.01471 \| 0.5609 \| 0.1792 \| 19 \| 14 \| 4.426 \| 50 \| |
| **Figure S5B: GFAP % area-Hippo** |
| \| Number of families \| 1 \|  \|  \|  \|  \|  \|  \|  \| \| --- \| --- \| --- \| --- \| --- \| --- \| --- \| --- \| --- \| \| Number of comparisons per family \| 3 \|  \|  \|  \|  \|  \|  \|  \| \| Alpha \| 0.05 \|  \|  \|  \|  \|  \|  \|  \| \|  \|  \|  \|  \|  \|  \|  \|  \|  \| \| Tukey's multiple comparisons test \| Mean Diff. \| 95.00% CI of diff. \| Significant? \| Summary \| Adjusted P Value \|  \|  \|  \| \|  \|  \|  \|  \|  \|  \|  \|  \|  \| \| Cont vs. Syn^A35T^/NOS^+/+^ \| -5.15 \| -9.19 to -1.11 \| Yes \| ** \| 0.0094 \| G-H \|  \|  \| \| Cont vs. SynA^35T^/NOS-^/-^ \| 0.6077 \| -3.814 to 5.029 \| No \| ns \| 0.9409 \| G-I \|  \|  \| \| Syn^A35T^/NOS^+/+^ vs. SynA^35T^/NOS-^/-^ \| 5.758 \| 1.287 to 10.23 \| Yes \| ** \| 0.0086 \| H-I \|  \|  \| \|  \|  \|  \|  \|  \|  \|  \|  \|  \| \|  \|  \|  \|  \|  \|  \|  \|  \|  \| \| Test details \| Mean 1 \| Mean 2 \| Mean Diff. \| SE of diff. \| n1 \| n2 \| q \| DF \| \|  \|  \|  \|  \|  \|  \|  \|  \|  \| \| Cont vs. Syn^A35T^/NOS^+/+^ \| 1.916 \| 7.066 \| -5.15 \| 1.669 \| 19 \| 18 \| 4.363 \| 47 \| \| Cont vs. SynA^35T^/NOS-^/-^ \| 1.916 \| 1.308 \| 0.6077 \| 1.827 \| 19 \| 13 \| 0.4704 \| 47 \| \| Syn^A35T^/NOS^+/+^ vs. SynA^35T^/NOS-^/-^ \| 7.066 \| 1.308 \| 5.758 \| 1.847 \| 18 \| 13 \| 4.408 \| 47 \| |
